# Discovery of Potent EGFR Inhibitors With *6-Arylureido-4-anilinoquinazoline* Derivatives

**DOI:** 10.3389/fphar.2021.647591

**Published:** 2021-05-26

**Authors:** Meng Li, Na Xue, Xingang Liu, Qiaoyun Wang, Hongyi Yan, Yifan Liu, Lei Wang, Xiaowei Shi, Deying Cao, Kai Zhang, Yang Zhang

**Affiliations:** ^1^ Department of Medicinal Chemistry, Hebei Medical University, Shijiazhuang, China; ^2^ Department of Pharmaceutical Engineering, Hebei Chemical and Pharmaceutical College, Shijiazhuang, China

**Keywords:** EGFR, anti-proliferative bioactivities, enzyme activity inhibition assay, molecular docking, molecular dynamic simulation

## Abstract

According to the classical pharmacophore fusion strategy, a series of *6-arylureido-4-anilinoquinazoline* derivatives (***Compounds 7a***–***t***) were designed, synthesized, and biologically evaluated by the standard CCK-8 method and enzyme inhibition assay. Among the title compounds, ***Compounds 7a***, ***7c***, ***7d***, ***7f***, ***7i***, ***7o***, ***7p***, and ***7q*** exhibited promising anti-proliferative bioactivities, especially ***Compound 7i***, which had excellent antitumor activity against the A549, HT-29, and MCF-7 cell lines (IC_50_ = 2.25, 1.72, and 2.81 μM, respectively) compared with gefitinib, erlotinib, and sorafenib. In addition, the enzyme activity inhibition assay indicated that the synthesized compounds had sub-micromolar inhibitory levels (IC_50_, 11.66–867.1 nM), which was consistent with the results of the tumor cell line growth inhibition tests. By comparing the binding mechanisms of ***Compound 7i*** (17.32 nM), gefitinib (25.42 nM), and erlotinib (33.25 nM) to the EGFR, it was found that ***Compound 7i*** could extend into the effective region with a similar action conformation to that of gefitinib and interact with residues L85, D86, and R127, increasing the binding affinity of ***Compound 7i*** to the EGFR. Based on the molecular hybridization strategy, 14 compounds with EGFR inhibitory activity were designed and synthesized, and the action mechanism was explored through computational approaches, providing valuable clues for the research of antitumor agents based on EGFR inhibitors.

## Introduction

Malignant tumors are an extremely serious public health problem that has aroused worldwide attention, making the discovery of antitumor drugs a research hotspot (Kim et al., 2016; El-Sayed et al., 2018; Yang et al., 2019; Zhang et al., 2019). Among the therapeutic targets for cancer, the abnormal expression of the epidermal growth factor receptor (EGFR) is strongly associated with various malignancies such as breast, ovarian, non-small-cell lung, prostate, and colon cancers. The EGFR has been confirmed to be closely related to tumor growth, progression, metastasis, and the poor prognosis of cancer patients, prompting extensive studies on the EGFR signaling pathway (Bishayee, 2000; Bridges, 2001; Ogiso et al., 2002; Hirsch et al., 2003; Bazley and Gullick, 2005; Allam et al., 2020; Han et al., 2020). Increasing evidence has shown that EGFR inhibitors have great potential in the treatment of tumors, especially non-small-cell lung cancer, hepatocellular carcinoma, and pancreatic cancer, which has inspired a research boom based on the design and synthesis of EGFR inhibitors (Umekita et al., 2000; Elgazwy et al., 2013; Mghwary et al., 2019; Sever et al., 2019; Sun et al., 2019; Zhu et al., 2019; Zhang et al., 2020).

**FIGURE 1:**
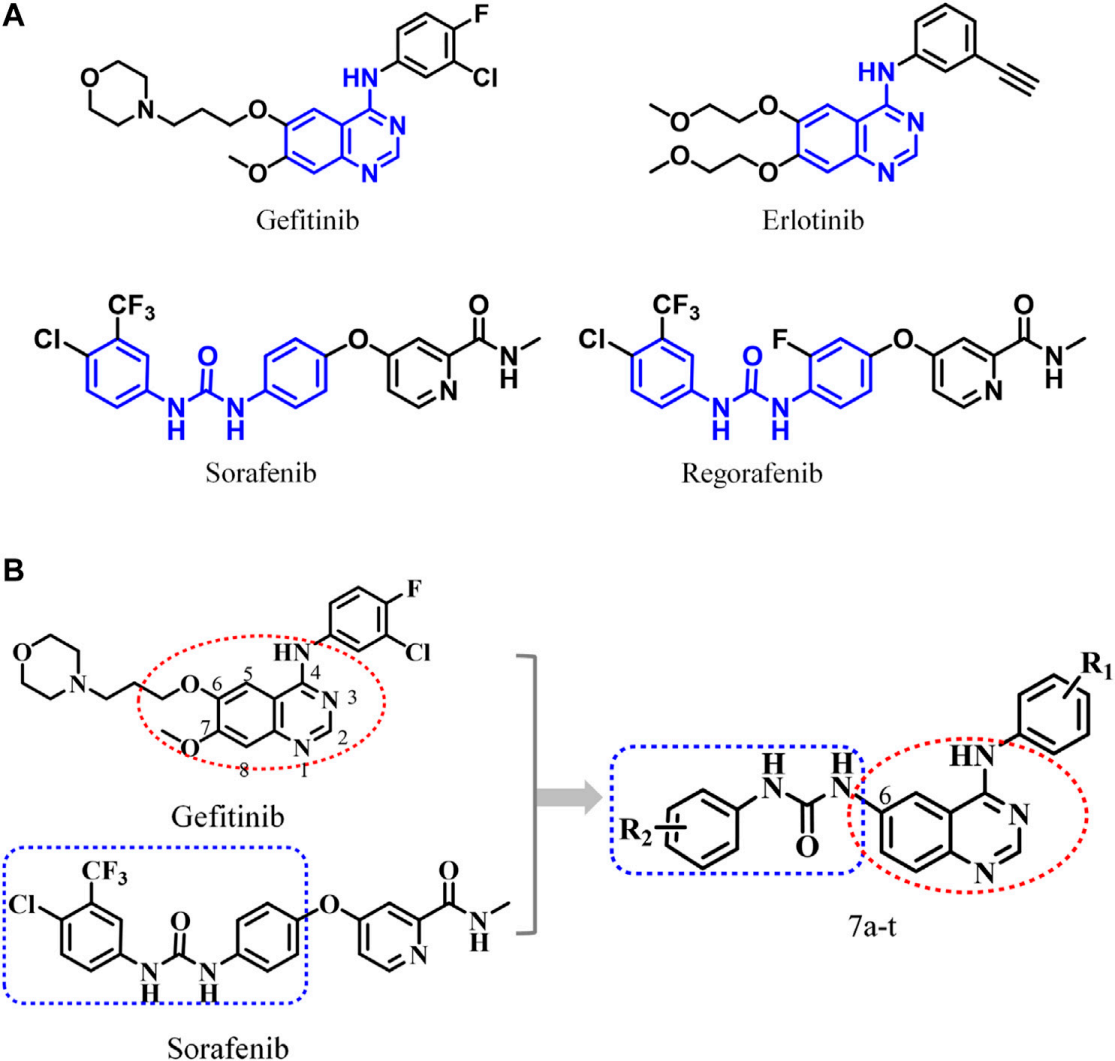
**(A)** Antitumor drugs with 4-anilinoquinazoline and diaryl urea structural fragments; **(B)** design scheme of 6-ureido-4-anilinoquinazoline derivatives.

Among the EGFR-based FDA-approved drugs, quinazoline derivatives have been recognized as selective and potent inhibitors (Figure 1A), exerting remarkable antitumor activity (Simon et al., 2003; Smith, 2005). This quinazoline scaffold can offer hydrogen bond acceptors and hydrogen bond donors and has a higher probability of forming hydrogen bonds with the receptor, providing stronger binding affinity with the target and ensuring promising enzyme inhibitory activity (Stamos et al., 2002; Vema et al., 2003; Mowafy et al., 2013; Rao et al., 2013; Yu et al., 2017; Zhang et al., 2017; Wei et al., 2019; Le et al., 2020). Moreover, the aryl urea structure with two hydrogen bond donors has received extensive attention. The compounds containing such moiety can enhance their interactions with the targets and have promising target affinity and strong inhibitory activities against KDR, B-Raf, RAF-1, PDGFR-β, C-KIT, FIT-3, and other kinases (Wan et al., 2004; Murphy et al., 2006; Eisen et al., 2011; Garofalo et al., 2012; Dungo and Keating, 2013; Ravez et al., 2015). Due to economic factors and effectiveness, molecular hybridization has become one of the most popular drug design strategies via combining two different active pharmacophores with or without the help of a linker, which is conducive to the rapid discovery of target compounds (Wan et al., 2004; Wilhelm et al., 2004; Wang et al., 2016; Kumar et al., 2017; Zheng et al., 2017; Ju et al., 2018; Asquith et al., 2019; Wei et al., 2019; Allam et al., 2020; Fu et al., 2020; Gontijo et al., 2020; Reddyrajula et al., 2019). Accordingly, potent EGFR inhibitors were designed and synthesized by ingeniously combining the above two advantageous skeletons (Figure 1B).

In this study, 20 6-arylureido-4-anilinoquinazoline derivatives were synthesized, and the bioactivities of the target compounds were evaluated by the cell anti-proliferative assay, EGFR preliminary screening test, EGFR kinase inhibition assay *in vitro*, and so on. Furthermore, comprehensive computational approaches were applied to compare the inhibitory mechanisms of the most promising and representative synthesized compound (***Compound 7i***) and the two approved drugs (gefitinib and erlotinib) at the molecular level. Finally, we found that the 4-anilinoquinazoline scaffold was an indispensable fragment, which interacted with most of the identified key residues with a relatively high energy contribution and guaranteed the basic binding affinity. In addition, the aryl urea fragment of ***Compound 7i*** could extend into the effective region and interact with residues L85, D86, and R127, presenting a similar action conformation to that of gefitinib in the EGFR. This research reasonably fused the above-mentioned dominant structures and discovered a series of inhibitors with enzymatic activity, providing valuable clues for the design of antitumor drugs based on the EGFR.

## Experimental Section

### Materials and Instruments

A Bruker AV-400 spectrometer was applied to record the ^1^H NMR and ^13^C NMR spectra of the synthesized compounds dissolved in DMSO-*d*_*6*_ solution, and all chemical shifts were reported in ppm (δ). Infrared (IR) spectra of the target compounds were determined with a SHIMADZU FTIR-8400S spectrometer (KBr disks). Mass spectra were obtained on a 3200 QTRAP and Triple Q-TOF 5600+ high resolution mass spectrometer (AB/SCIEX). Melting points were measured with an M-560 MP apparatus (BUCHI) without correction. All chemical reactions were monitored by thin-layer chromatography (TLC) on silica gel (purchased from Yantai Xinnuo Chemical Plant), and the products were visualized with an ultraviolet lamp (254 and 365 nm).

All reagents and solvents met the criteria of analytical reagents before use. All cancer cell lines (A549, human non-small-cell lung cell line; HT-29, human colonic adenocarcinoma cell line; MCF-7, human breast cancer cell line) were purchased from *Stem Cell Bank, Chinese Academy of Sciences*. RPMI 1640 (Roswell Park Memorial Institute 1640), DMEM (Dulbecco’s modified Eagle’s medium), and FBS (fetal bovine serum) were obtained from *Corning* (Jiangsu, China). ADP-Glo^™^ kinase assay kits were provided by *Promega (Beijing) Biotech Co., Ltd.* (Beijing, China). All kinase subtypes were supplied by *SignalChem* (BC, Canada).

### Chemistry

**FIGURE 2:**
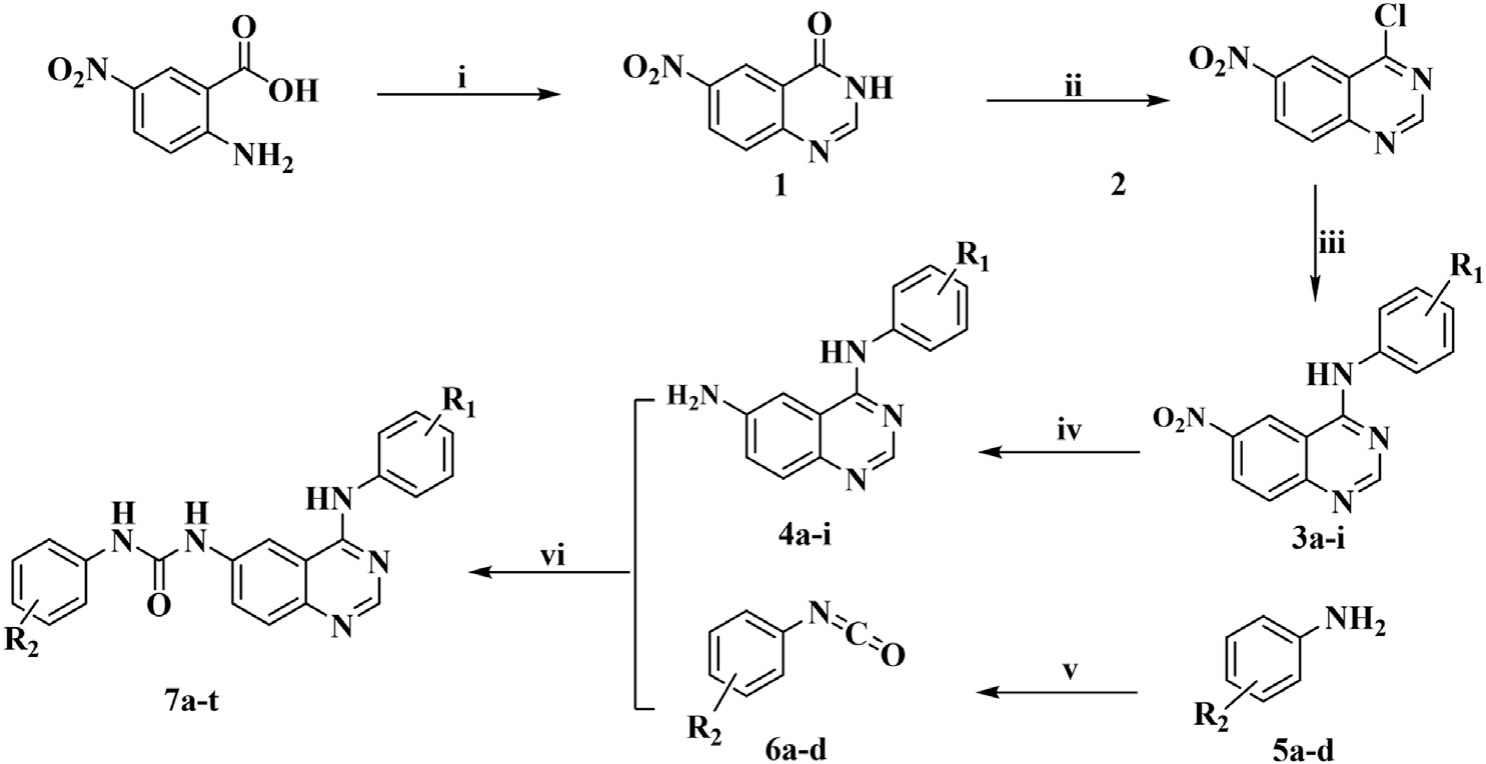
Synthetic route of the target ***Compounds 7a***–***t***.

The title compounds were obtained based on the synthetic route shown in Figure 2. Six aromatic ureido-4-anilinoquinazoline derivatives were synthesized by methods reported previously, mainly consisting of six experimental steps, and the whole synthetic process is described here represented by ***Compound 7i***. The purity of all the target compounds was determined by liquid-mass spectrometry. The synthesis and characterization of the other target compounds were given in *supplementary data*.

#### Synthesis of 6-Nitroquinazolin-4(3H)-one (**1**)

The mixture of 2-amino-4-nitrobenzoic acid (7.28 g, 40.0 mmol) and formamide (60 ml) was stirred at 150°C for 16 h, and the reaction process was monitored by TLC. Then, the mixture was cooled to room temperature, and the reaction solution was removed by filtration. After that, the product was purified by washing with isopropanol and dried to obtain ***Compound 1***. Yield: 51.6%; m.p. 285.0–286.0 °C; ^1^H NMR (400 MHz-DMSO-*d*_*6*_): δ 12.809 (brs, 1H), 8.791–8.784 (d, *J* = 2.8 Hz, 1H), 8.551–8.529 (dd, *J* = 2.4 Hz, 8.8 Hz, 1H), 8.314 (s, 1H), 7.866–7.843 (d, *J* = 9.2 Hz, 1H). MS (ESI^−^) m/z 190.0 (M-H)^−^.

#### Synthesis of 4-Chloro-6-nitroquinazoline (**2**)

***Compound 1*** was added to 23 ml of thionyl chloride for the chlorination reaction with DMF as the catalyst, and it should be noted that the reaction system was heated to reflux for 2.5 h until the solution was clear. Afterward, the solution was cooled to room temperature, and the solvent was vacuum distilled. Then, the reaction residues were diluted with CH_2_Cl_2_ and concentrated into a yellow solid (***Compound 2***), which was used directly in the next step without further purification. m.p. 130°C; ^1^H NMR (400 MHz-DMSO-*d*_*6*_): δ 8.78 (d, *J* = 2 Hz, 1H), 8.555 (dd, *J* = 6.7 Hz, 2 Hz, 1H), 8.432 (1H, s), 7.833 (d, *J* = 6.7 Hz, 1H). MS (ESI^−^) m/z 208.2 (M-H)^−^.

#### Synthesis of N-(3-Bromophenyl)-6-nitroquinazolin-4-amine (**3a**)

***Compound 2*** (2.65 g, 11.8 mmol) was dissolved in isopropanol, and 3-bromoaniline (2.41 g, 14.1 mmol) was added to the above solution at room temperature, which was heated to reflux. The progress of reaction was monitored by TLC until completion, and then the mixture was cooled to room temperature. Afterward, the resultant precipitate was collected by filtration and washed with isopropanol to obtain the yellow solid ***Compound 3a*** (2.01 g, 5.8 mmol) after drying. Yield: 85.2%; m.p. 287.8–289.6°C; ^1^H NMR (400 MHz-DMSO-*d*_*6*_): δ 12.088–12.067 (brs, 1H), 9.921–9.917 (d, *J* = 1.6 Hz, 1H), 8.999 (s, 1H), 8.783–8.755 (dd, *J* = 2.0, 9.2 Hz, 1H), 8.200–8.177 (d, *J* = 9.2 Hz, 1H), 7.778–7.285 (m, 4H). MS (ESI^+^) m/z 347.2 (M + H)^+^.

#### Synthesis of N^4^-(3-Bromophenyl)quinazoline-4,6-diamine (**4a**)

***Compound 3a*** (1.69 g, 4.9 mmol) and stannous chloride (4.42 g, 19.6 mmol) dissolved in ethyl acetate (49 ml) were stirred and refluxed for 1 h, and then the reaction system was cooled to room temperature. The solid precipitate was filtered under vacuum through Celite and washed with ethyl acetate (100 ml × 3), and the obtained aqueous layer was neutralized with saturated sodium carbonate solution (pH = 7). The aqueous phase was extracted with ethyl acetate (2 × 50 ml), and the resulting ethyl acetate solution was added to the organic phase previously separated, which was further concentrated under reduced pressure to obtain ***Compound 4a*** (1.23 g, 79.9%, yellow solid). Yield: 55.1%; m.p. 191.7–193.6°C; ^1^H NMR (400 MHz-DMSO-*d*_*6*_): δ 9.455 (s, 1H), 8.368 (s, 1H), 7.955–7.810 (m, 2H), 7.707–7.527 (m, 3H), 7.366–7.362 (d, *J* = 1.6 Hz, 1H), 7.287–7.265 (d, *J* = 8.8 Hz, 1H), 5.629 (s, 2H). MS (ESI^+^) m/z 316.3 (M + H)^+^.

#### Synthesis of 1-Chloro-4-isocyanato-2-(trifluoromethyl)benzene (**6b**)

4-Chloro-3-(trifluoromethyl)aniline (15 g, 76.7 mmol) dissolved in triethylamine (16.3 g, 161.0 mmol) was added dropwise to the solution of triphosgene (9.19 g, 30.0 mmol) in dichloromethane (150 ml). The obtained mixture was stirred at room temperature for 1 h and then refluxed for 3 h. Then, the reaction system was cooled to room temperature and concentrated to obtain an oily substance, which was further purified by vacuum distillation to obtain ***Compound 6b*** (colorless liquid, 6 g, 35.3%).

#### Synthesis of 1-(4-((3-Bromophenyl)amino)-3,4-dihydroquinazolin-6-yl)-3-(4-chloro-3-(trifluoromethyl)phenyl) Urea (**7i**)

Anhydrous acetonitrile was added to ***Compound 6b***, followed by the addition of ***Compound 4a***. The reaction system was stirred for 3 h and then filtered to obtain a solid product, which was rinsed with anhydrous acetonitrile and dried to obtain the final ***Compound 7i*** (1.21 g, 88.9%, 5.8 mmol). m.p. 268.0–268.9°C; compound purity: 95.413%; aqueous solubility: soluble; IR (KBr) γ/cm^−1^: 3,319, 3,065, 3,015, 1,707, 1,614, 1,572, 1,549, 1,483, 1,431, 1,323, 1,252, 1,204, 1,122, 1,030, 833, 773, 742, 679 cm^−1^; ^1^H NMR (400 MHz-DMSO-*d*_*6*_): δ 9.883 (s, 1H), 9.396 (s, 1H), 9.169 (s, 1H), 8.578–8.544 (m, 2H), 8.198–8.193 (d, *J* = 2.0 Hz, 2H), 7.889–7.862 (m, 2H), 7.803–7.781 (d, *J* = 8.8 Hz, 2H), 7.714–7.708 (d, *J* = 2.4 Hz, 1H), 7.692–7.636 (m, 2H), 7.377–7.288 (m, 2H); ^13^C NMR (100 MHz-DMSO-*d*_*6*_): δ 157.06, 152.65, 152.49, 146.00, 141.13, 139.19, 137.16, 132.04, 130.28, 128.46, 126.88, 126.57, 125.84, 124.31, 124.15, 123.17, 122.51, 121.43, 121.09, 120.90, 116.88, 116.82, 115.53, 110.79; HRMS (ESI): m/z calculated for (C_22_H_14_BrClF_3_N_5_O + H)^+^: 536.0100; found: 536.0115.

### Biological Assay

#### Anti-Proliferative Assay

The designed 4-anilinoquinazoline derivatives were evaluated for their antitumor bioactivities *in vitro* against three human tumor cell lines (A549, HT-29, and MCF-7) using the standard CCK-8 method. The CCK-8 method is based on WST-8 dye, 2-(2-methoxy-4-nitrophenyl)-3-(4-nitrophenyl)-5-(2,4-disulfophenyl)-2H-tetrazolium. WST-8 can be bio-reduced by dehydrogenases in mitochondria into orange water-soluble formazan products. Briefly, selected cancer cells in the logarithmic growth phase (1 × 10^4^) were inoculated in 96-well plates and further cultured in the incubator for 24 h. First, the target compounds were diluted to different concentrations with culture medium with each dilution gradually diluted from the highest concentration solution. Second, the original culture medium was removed after the cells were completely adherent to the wall, and the compounds at various concentrations prepared above were added. In this experiment, a control group and a blank group were set: the former was treated with 0.1% DMSO solution, and the latter was treated with only the culture medium. Finally, 10 μl of CCK-8 detection solution was added to each well followed by culture in an incubator for 1–3 h. A microplate reader was adopted to record the absorbance at 450 nm, and in this assay, gefitinib, erlotinib, and sorafenib were used as the reference drugs.

#### Kinase Inhibition Assay

The EGFR kinase inhibition activities of the target compounds were evaluated by the ADP-Glo^™^ assay. The ADP-Glo^™^ kinase detection kit is a luminescence kinase detection approach that detects the amount of ADP produced by the kinase reaction. After ADP is converted into ATP, ATP can be used as the substrate of the luciferase-catalyzed reaction to generate an optical signal, which is positively correlated with kinase activity. An ADP-Glo^™^ kinase detection kit can detect the activities of almost all enzymes that can produce ADP, and the concentration of ATP can be as high as 1 mmol. The target compounds were formulated into solutions of different concentrations (0.46, 1.37, 4.12, 12.35, 37.04, 111.11, 333.33, and 1,000 nm), and three independent experiments were performed for each group. The following components were added to the 384-well plate: 5 μl of kinase buffer containing 20 μmol ATP and 2 μmol PIP2 (25 mmol 3-morpholinopropanesulfonic acid, 12.5 mmol β-glycerophosphoric acid, 5 mmol EGTA, 2 mmol EDTA, and 0.25 mmol DTT); 2 μl (50 ng/ml) of EGFR kinase; and 2.5 μl of dimethyl sulfoxide solution containing different concentrations of the test compound. The above-prepared systems were sealed and incubated at room temperature for 2 h, and 5 μl of ADP-GLO was added to terminate the kinase reaction. Then, the culture plate was sealed and incubated in a thermostatic oscillator for 40 min to fully consume the remaining ATP. The luciferase/luciferin reaction was adopted to determine the newly generated ATP level. The signals of ADP/ATP varied according to the inhibitory effects of the target compounds, the luminescence values of each well were measured with an *EnVision 2014* plate counter, and the data were further converted into IC_50_ values using *GraphPad Prism 5.0* software.

Moreover, to further determine the targets of the synthetic compounds, ***Compound 7i*** was selected as the representative molecule for evaluation by kinase spectrum assays according to the above method. In this study, six kinds of promising protein kinase targets for cancer therapy (including 30 kinases) were applied: 1) *tyrosine kinases* (TKs) (Pflug et al., 2001; Steelman et al., 2004; Wilhelm et al., 2006; Oneyama et al., 2008; Bae and Schlessinger, 2010; Zhao and Guan, 2011; Sánchez-Bailón et al., 2012; Liu et al., 2016; Leick and Levis, 2017; Huang et al., 2018; Kassouf et al., 2019)—Ab1, Csk, FAK, FGFR2, HER4, EGFR, LCK, SRC, Syk, TRKA, FLT3, KDR, JAK3; 2) *STE kinases* (Malinin et al., 1997)—NIK, ASK1; 3) *AGC kinases* (Leroux et al., 2018)—PKCa, ROCK1, PDK1, PKACα; 4) *CAMK kinases* (Beullens et al., 2005; Kobayashi et al., 2006; David et al., 2016)—Chk1, MAPKAPK2, MELK, CAMK2α; 5) *CMGC kinases* (Kim et al., 2001; Mihara et al., 2001)—CDK2, CLK3, JNK1, ERK2, GSK3β; and 6) *TKL kinase*s (Schulze et al., 2001; Srivastava et al., 2012)—IRAK4, *B-raf*. According to the results, ***Compound 7i*** significantly inhibited the EGFR significantly (enzyme activity = 10.52%). In addition, ***Compound 7i*** did not show significant inhibitory activity against other kinases (enzyme activity > 50%).

### Molecular Simulation Studies

#### Molecular Docking

Here, the *LibDock* module in *Discovery Studio 2020 (DS 2020)* was adopted to perform molecular docking. First, the docked ligands were constructed by *ChemBioDraw Ultra 14.0* and saved as an *.sdf file type and were further processed with the “*Prepare Ligands and Minimize Ligands*” modules in DS 2020, including changing ionization, generating tautomers, generating isomers, fixing bad valencies, generating 3D coordinates, and energy minimization using the *CHARMm* force field. Second, the crystal structure of the EGFR was downloaded from the Protein Data Bank and prepared by the following two steps: 1) clean protein and 2) prepare protein. The preprocessed receptor generated the docking site at the original ligand site for subsequent molecular docking, and the default parameters were used for docking calculations. Then, the ligand conformation, interactions between the ligand and the receptor, and the docking score were used to evaluate the docking poses, and the appropriate conformations were selected for molecular dynamics simulations.

#### Molecular Dynamics Simulations

Before performing MD simulations, three PDB files were prepared, including the receptor–ligand complex, the receptor, and the ligand. *Gaussian 09* at the HF/6-31G^*^fnlowast level was applied to optimize the geometries and calculate the electrostatic potential of the ligands and generate the *.log files, which were used to create *.frcmod and mol_2_ formats via an *antechamber* model in *AMBERTOOLS16*. Then, the coordinate files (.inpcrd) and topology files (.prmtop) of the receptor–ligand complex, receptor, and ligand were generated using the *LEaP* module with the corresponding force field (*ff14SB53* for the protein, *general AMBER force field* (*gaff*) for the ligands). Then, MD production was conducted using GPU-accelerated PMEMD (three pieces of the NVIDIA Tesla P100 PCIe graphic card), and 100 ns of production simulations were carried out for the three studied systems in NPT ensembles at 310 K and 1 atm.

## Results and Discussion

### Chemistry

Twenty target compounds were synthesized based on the synthetic route shown in Figure 2, and the yield of intermediates was relatively high in each step. Six aromatic ureido-4-anilinoquinazoline derivatives were synthesized by the methods reported previously, mainly consisting of six experimental steps: i) ***Compound 1*** was obtained by cyclization of 2-amino-4-nitrobenzoic acid with formamide, with the yield of 51.6%. ii) ***Compound 2*** was obtained by the chlorination reaction of ***Compound 1*** with thionyl chloride. iii) ***Compound 2*** underwent nucleophilic substitution reaction with the corresponding aniline without purification to acquire ***Compounds 3a***–***i*** (yield: 72.0–98.5%). iv) The intermediate obtained in the above step was reduced under the action of stannous chloride to give ***Compounds 4a***–***i***. v) Aniline with different substituents reacted with triphosgene to give isocyanates (***Compounds 6a***–***d***). It is worth noting that the synthesis and post-treatment process of isocyanate were relatively complicated, and the selected reagent should be anhydrous. In addition, the purity of the isocyanate directly affected the yield of the target compounds, and in this study, vacuum distillation was used to purify the isocyanate, thus reducing the generation of impurities and improving the yield of the final compounds. vi) ***Compounds 4a***–***i*** and ***6a***–***d*** underwent nucleophilic addition reaction to generate the title ***Compounds 7a***–***t*** (yield: 45.0–87.6%), and all the target compounds and important intermediate were structurally characterized by IR, HRMS, ^1^H NMR, and ^13^C NMR. Moreover, the target compounds had good water solubility and have been tested by liquid-mass spectrometry.

### Anti-Proliferative Assay

The standard CCK-8 method was applied to evaluate the anti-proliferative activities of the target compounds against A549, HT-29, and MCF-7 cells with gefitinib, erlotinib, and sorafenib as the positive references, and the results are shown in Table 1. As illustrated in Table 1, the synthesized compounds showed varying degrees of antitumor activity. ***Compounds 7a***–***h*** with -H as the R_2_ group had moderate anti-proliferative effects against all the three tumor cell lines. Among them, in the compounds with -Br as the R_1_ group, the biological activities were relatively good, especially in ***Compounds 7a*** and ***7c***. When the -CH_3_ group was introduced into the R_1_ site, the inhibitory activities of the corresponding compounds decreased, especially with the introduction of the 2,3-CH_3_ group (***Compound 7h*** > 50 μm). When R_2_ was 3-CF_3_ and 4-Cl, ***Compounds 7i***–***n*** had strong antitumor activities (IC_50_, 1.72–5.68 μm) against the selected cell lines, especially ***Compound 7i***, which were better than other derivatives as well as the positive controls (gefitinib, erlotinib, and sorafenib: 1.84–23.22 μm). When the R_1_ group was substituted by -Br, the compound exerted promising antitumor activity, and the 3-Br substituent was superior to the 4-Br substituent. ***Compounds 7o***–***t*** with 4-F or 4-OCH_3_ as the R_2_ group showed no obvious activity against all the tested cell lines and only showed weak anti-proliferation effects. Through the comprehensive evaluation of the R_1_ group and the R_2_ group, it can be found that Br on the R_1_ group and 3-CF_3_ and 4-Cl on the R_2_ group were essential for maintaining the activity of the target compounds.

**TABLE 1:** Cellular results for the ***Compound 7*** series.

Compound	R_1_	R_2_	Proliferative inhibition (IC_50_, μM)^a^		
			A549	HT-29	MCF-7
**7a**	3-Br	H	5.81 ± 0.84	4.66 ± 0.56	15.90 ± 2.28
**7b**	4-OCH_3_	H	39.55 ± 0.45	9.13 ± 0.48	12.88 ± 1.31
**7c**	2-F, 4-Br	H	6.16 ± 0.45	5.92 ± 1.04	8.57 ± 0.27
**7d**	3-Cl, 4-F	H	38.65 ± 0.21	39.23 ± 2.01	44.23 ± 0.97
**7e**	2,4,6-CH_3_	H	39.12 ± 1.10	9.58 ± 0.21	12.87 ± 0.60
**7f**	4-F	H	>50	48.26 ± 0.78	44.27 ± 1.12
**7g**	4-CH_3_	H	>50	43.14 ± 0.29	42.23 ± 1.32
**7h**	2,3-CH_3_	H	>50	>50	>50
**7i**	3-Br	3-CF_3_, 4-Cl	2.25 ± 0.08	1.72 ± 0.49	2.81 ± 0.09
**7j**	4-OCH_3_	3-CF_3_, 4-Cl	2.55 ± 0.31	3.41 ± 1.27	5.68 ± 0.32
**7k**	4-Br	3-CF_3_, 4-Cl	2.79 ± 0.44	2.30 ± 0.50	3.54 ± 0.07
**7l**	4-F	3-CF_3_, 4-Cl	3.19 ± 0.28	3.03 ± 1.19	3.95 ± 1.26
**7m**	4-CH_3_	3-CF_3_, 4-Cl	5.60 ± 0.31	2.61 ± 1.25	5.53 ± 0.39
**7n**	2,3-CH_3_	3-CF_3_, 4-Cl	2.67 ± 0.76	2.67 ± 1.02	3.11 ± 0.30
**7o**	4-CH_3_	4-F	35.28 ± 0.57	7.83 ± 0.97	33.67 ± 10.08
**7p**	2,3-CH_3_	4-OCH_3_	9.34 ± 0.73	11.14 ± 0.39	40.23 ± 2.13
**7q**	4-Br	4-OCH_3_	5.21 ± 0.30	36.96 ± 0.17	17.87 ± 0.89
**7r**	4-F	4-OCH_3_	>50	48.21 ± 0.23	46.14 ± 1.98
**7s**	4-CH_3_	4-OCH3	>50	46.23 ± 0.57	47.56 ± 0.76
**7t**	4-OCH_3_	4-OCH_3_	>50	48.29 ± 1.90	>50
Gefitinib	—	—	14.75 ± 2.48	8.06 ± 0.22	15.68 ± 0.25
Erlotinib	—	—	23.22 ± 0.51	22.75 ± 1.12	11.42 ± 0.43
Sorafenib	—	—	1.84 ± 0.17	2.27 ± 0.43	3.47 ± 0.39

a The values are the mean ± SD of at least three independent experiments.

### EGFR Inhibitory Activity

**FIGURE 3:**
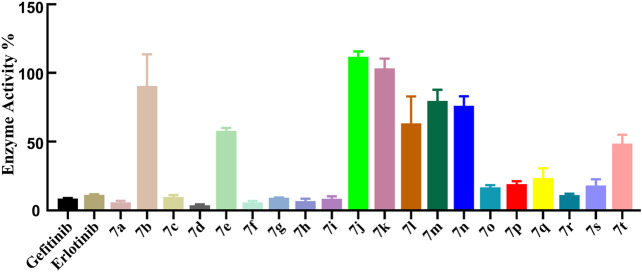
EGFR enzyme activities of the ***Compound 7*** series at 1 μm.

The ADP-Glo™ approach was adopted to further evaluate the kinase inhibitory activities of the target compounds, and gefitinib and erlotinib as commonly clinical EGFR inhibitors were also used as positive controls. First, the EGFR preliminary screening test on ***Compounds 7a***–***t*** was carried out, and most of the tested compounds had relatively strong EGFR inhibitory activity (enzyme activity% ≤ 50%) at the concentration of 1 μm (Figure 3). The IC_50_ values of the compounds with better preliminary screening experimental results were further determined, and it was found that these compounds all showed promising enzyme inhibitory activities (ranging from 11.66 to 867.1 nm, Table 2). Compared with classic EGFR inhibitors, the stronger EGFR inhibitory activities of the designed compounds might be caused by the aryl urea group introduced by the C-6 position of the quinazoline scaffold. The introduction of the aryl urea group would enlarge the molecular framework and increase the possibility of interactions between the key amino acids in the active pocket and the designed compounds, thereby improving their binding affinity to the target.

**TABLE 2:** Enzymatic results for part of the ***Compound 7*** series

Compound	Enzymatic inhibition IC_50_ (nM)^a^	Compound	Enzymatic inhibition IC_50_ (nM)^a^
	EGFR		EGFR
**7a**	12.01 ± 0.20	**7o**	123.9 ± 1.09
**7c**	20.08 ± 2.21	**7p**	58.12 ± 1.99
**7d**	11.79 ± 0.36	**7q**	470.4 ± 1.28
**7e**	867.1 ± 1.12	**7r**	59.66 ± 1.31
**7f**	11.66 ± 0.79	**7s**	107.70 ± 2.64
**7g**	72.36 ± 1.05	**7t**	728.1 ± 1.65
**7h**	19.73 ± 1.87	Gefitinib	25.42 ± 0.85
**7i**	17.32 ± 0.54	Erlotinib	33.25 ± 1.02

a The values are the mean ± SD of at least two independent experiments.

It should be noted that, unlike the results of the cell anti-proliferation assay, the compounds with H as the R2 group showed better biological activities, especially ***Compounds 7a***, ***7c***, ***7d***, ***7f***, and ***7h*** (11.66–20.08 nm). In addition, the compounds with halogen atoms as the R_1_ group showed better bioactivities, especially ***Compounds 7d*** and ***7f***. In addition, the compounds showed moderate inhibitory activities with 4-OCH_3_ as the R_2_ group (58.12–728.10 nm). By comparing the biological evaluations, it could be concluded that the results of anti-cell proliferation of ***Compounds 7a***, ***7c***, and ***7i*** were consistent with those of enzyme inhibition assays, especially ***Compound 7i***, which was also the main reason that ***Compound 7i*** was selected as the representative molecule for subsequent molecular simulation. Furthermore, the inhibitory activities of ***Compound 7i*** against six kinds of protein kinases (including 30 protein kinases) were evaluated at a concentration of 1.0 μm. As shown in Table 3, ***Compound 7i*** showed excellent activity against EGFR protein kinase (EGFR enzyme activity = 10.52%) but no significant activity against the other protein kinases (>50% enzyme activity), indicating good kinase-targeting properties and the rationality of the design.

**TABLE 3:** Kinase spectrum test of ***Compound 7i*** at 1 μm.

Kinase	Enzyme activity %^a^	Kinase	Enzyme activity %^a^
Abl	94.51 ± 0.41	PKCa	77.27 ± 1.18
Csk	93.76 ± 4.09	ROCK1	78.70 ± 4.00
FAK	81.70 ± 2.75	PDK1	74.18 ± 0.62
FGFR2	91.07 ± 8.02	PKACα	86.02 ± 3.75
HER4	76.85 ± 0.33	Chk1	75.98 ± 6.72
EGFR	10.52 ± 0.26	MAPKAPK2	86.46 ± 7.38
LCK	86.84 ± 6.53	MELK	78.83 ± 6.55
SRC	72.10 ± 1.83	CAMK2α	72.67 ± 2.92
Syk	101.13 ± 9.99	CDK2	77.40 ± 6.84
TRKA	83.52 ± 3.13	CLK3	86.80 ± 1.90
FLT3	92.18 ± 15.36	JNK1	89.43 ± 2.48
KDR	79.66 ± 2.00	ERK2	97.31 ± 11.84
JAK3	82.49 ± 1.82	GSK3β	103.71 ± 1.75
NIK	76.97 ± 5.84	IRAK4	86.33 ± 0.56
ASK1	62.27 ± 3.02	B-raf	96.68 ± 4.29

a The values are the mean ± SD of at least two independent experiments.

### Molecular Simulation Studies

**FIGURE 4:**
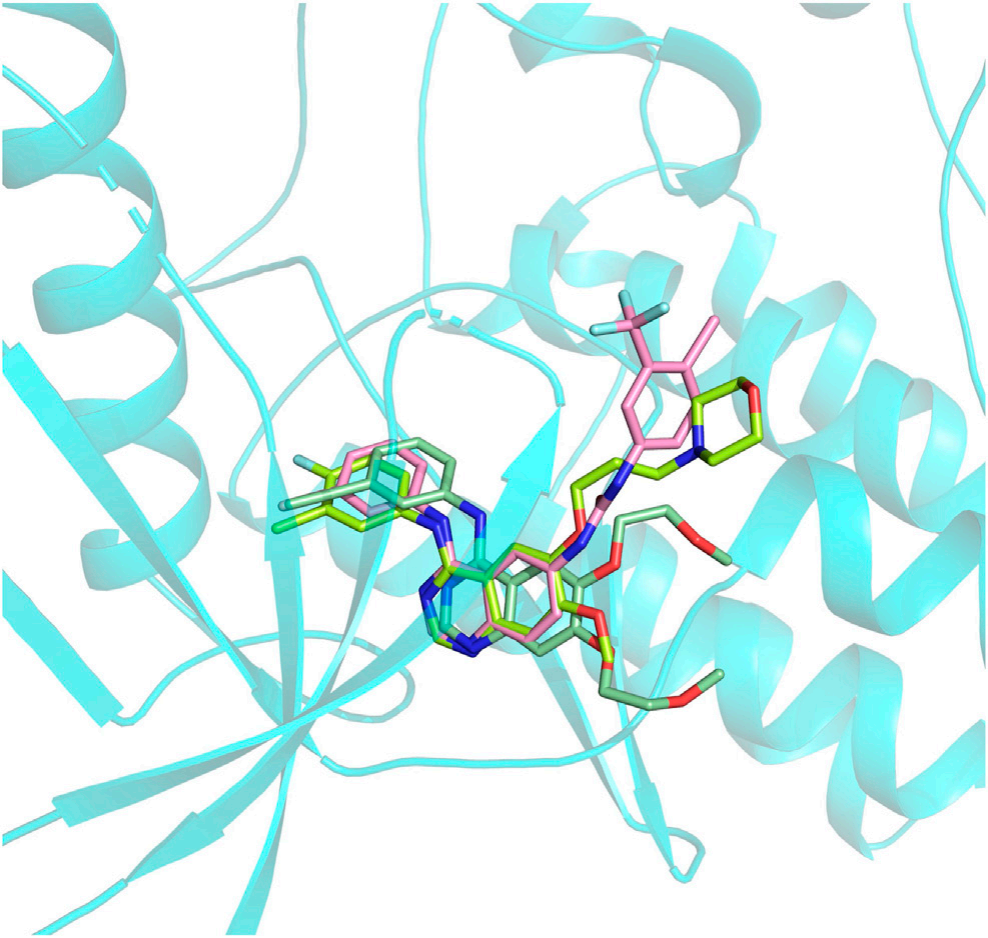
Alignment of initial docking poses of the three systems: erlotinib is marked in pale green, gefitinib is in lemon, and ***Compound 7i*** is in light pink.

In this section, ***Compound 7i*** was chosen as the representative ligand to explore the binding mechanism of such scaffolds bearing a 4-anilinoquinazoline moiety. First, the initial conformations of ***Compound 7i***, gefitinib, and erlotinib in the EGFR were constructed by molecular docking based on the crystal structure provided by the Protein Data Bank (PDB entry ID: 4WKQ). Second, the binding modes of the three selected ligands were illustrated through molecular dynamic simulation. Figure 4 shows that all docking poses were highly consistent spatially, especially the 4-anilinoquinazoline fragment surrounded by three β-sheets and two loop domains.

**FIGURE 5:**
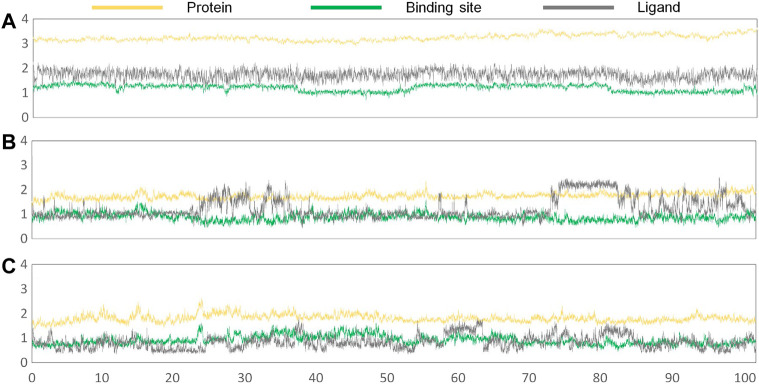
RMSD values of the protein receptor (yellow), ligand (deep gray), and binding sites (green) of the constructed systems as the function of time of the second simulation: **(A) *Compound 7i*** system; **(B)** gefitinib system; **(C)** erlotinib system.

Subsequently, the initial conformations obtained above were subjected to 100 ns of MD simulation, and the dynamic stability was evaluated by the root mean square deviation (RMSD) values. According to Figures 5A–C, all of the studied systems could reach dynamic equilibrium after 40 ns, especially the protein receptor and the residues consisting of the binding sites. Additionally, it should be noted that gefitinib and erlotinib had certain fluctuations during the MD process, which might be related to the saturated alkane in their molecular skeletons.

**FIGURE 6:**
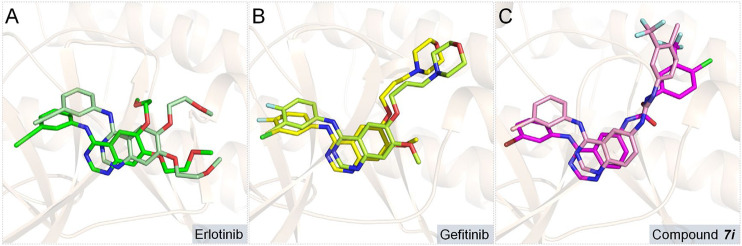
Superimposition of the initial and representative conformations of the three studied systems: **(A)** erlotinib system (representative conformation is colored in green; initial conformation is marked in pale green); **(B)** gefitinib system (representative conformation is colored in yellow; initial conformation is in limon); **(C) *Compound 7i*** system (representative conformation is in magenta; initial conformation is in light-pink).

In order to further assess the binding conformations of the three systems, the dynamic trajectories of the final 50 ns of the simulations were applied to calculate the binding free energies of the complexes. The binding free energies of the studied systems were −58.49, −54.35, and −53.53 kcal/mol for ***Compound 7i***, gefitinib, and erlotinib, respectively, which were consistent with their enzyme inhibitory activities (17.32, 25.42, and 33.25 nm, respectively). Then, the initial and representative conformations’ superposition analyses were carried out, and the results are shown in Figures 6A–C. In all research systems, the ligand conformations changed slightly at the binding sites in order to accommodate the binding of the compounds to the receptor.

**FIGURE 7:**
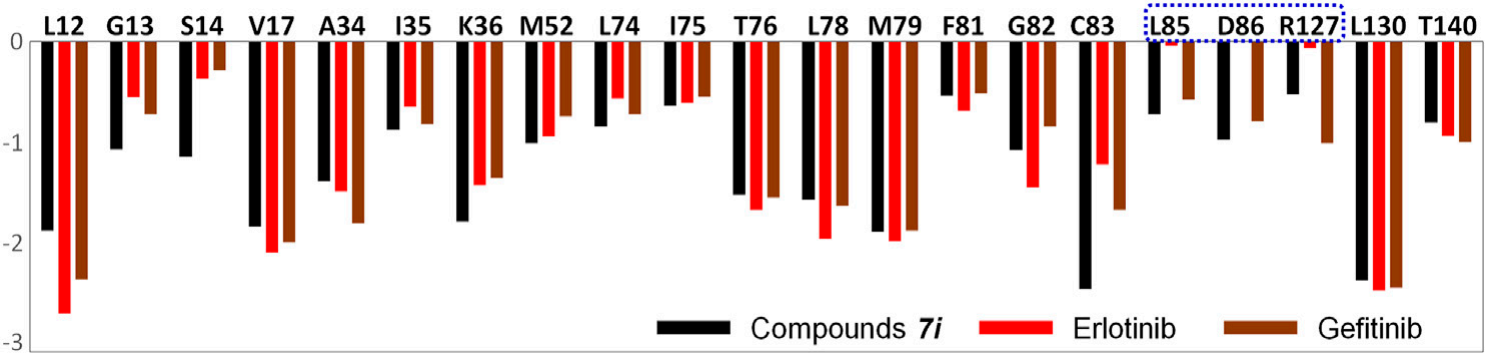
Per-residue binding free energy decomposition of all the studied systems: the residues with energy contribution (absolute value ≥ 0.1) are selected.

Furthermore, the amino acid energy contribution was adopted to compare the binding modes of the studied systems. Figure 7 shows that the majority of the amino acids had a small difference in the energy contribution to ligand binding. By mapping these residues into the three-dimensional space, it was found that such residues were generally located in the above-mentioned domains: three β-sheets and two loop domains. Moreover, only three amino acids showed significant differences in their energy contributions, namely, L85, D86, and R127, which favored the binding of gefitinib and ***Compound 7i.***

**FIGURE 8:**
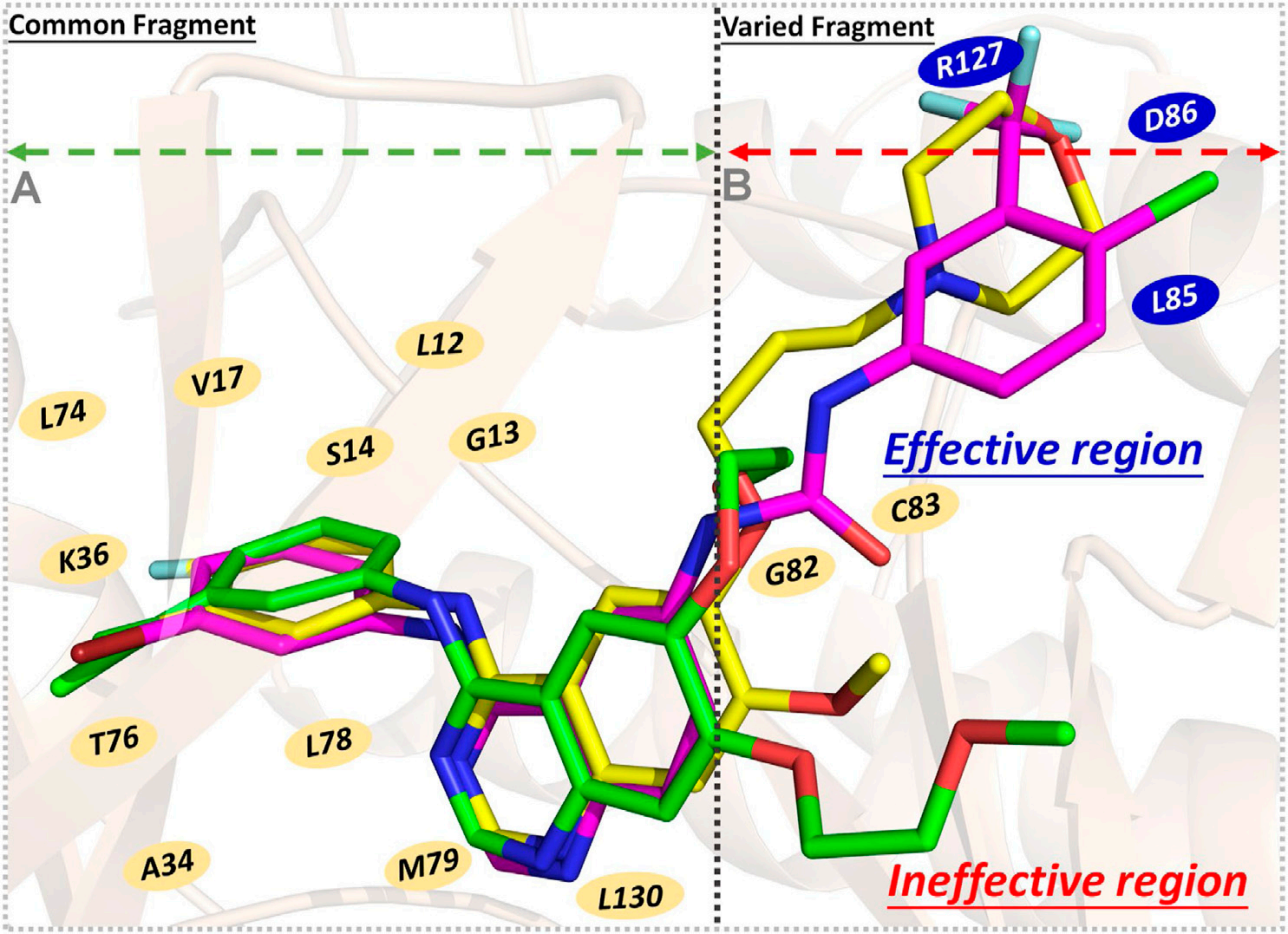
Schematic diagram of three ligands’ binding modes in the EGFR - gefitinib is in yellow, erlotinib is in green, and ***Compound 7i*** is in magenta: **(A)** Region A, the residues interacting with the common fragment; **(B)** Region B, the residues interacting with the varied fragment.

Finally, the binding modes of three ligands in the EGFR were determined. According to Figure 8A, residues with significant energy contributions were mainly located in region A, such as L12, G13, S14, L74, and T76, which interacted strongly with the 4-anilinoquinazoline scaffold and played important roles in the stable binding of the studied ligands to the EGFR and guaranteed the enzyme inhibitory activities at the nanomolar level. In addition, from a binding free energy (BFE) perspective, we found that the BFE of erlotinib was lower than that of gefitinib and ***Compound 7i***, which may be mainly caused by the differences in the molecular skeletons. The remaining fragments of gefitinib and ***Compound 7i*** were found to extend into region B and interact with L85, D86, and R127 (Figure 8B), but erlotinib could not interact with this region due to the flexibility of the methoxy–ethoxy groups, resulting in the relatively weak enzyme inhibitory activity of erlotinib (33.25 nm).

Therefore, the 4-anilinoquinazoline scaffold was an indispensable fragment of this series of EGFR inhibitors, ensuring the considerable binding free energies of their interactions with the receptor, and should be retained to a greater extent in subsequent molecular design. Moreover, in the process of designing novel EGFR inhibitors, it was necessary to pay attention to the interactions between the skeleton and the residues located in region B (especially L85, D86, and R127), which were beneficial to enhance the ligand’s binding affinity. Therefore, the length and rigidity of the molecular scaffold should also be considered, allowing extension into the above-mentioned region.

## Conclusion

In summary, 20 6-ureido-4-anilinoquinazoline derivatives were designed and synthesized, and their biological activities were evaluated at the cellular and kinase levels. According to the experimental results, the majority of the synthesized compounds targeted the EGFR and showed good anti-proliferative activities against A549, HT-29, and MCF-7 cells, especially ***Compound 7i***. In addition, the action mechanisms of ***Compound 7i***, gefitinib, and erlotinib were compared by molecular docking and MD simulations, and it was found that the 4-anilinoquinazoline scaffold was an indispensable fragment in this series of EGFR inhibitors, guaranteeing the considerable binding free energy with the receptor. Moreover, the introduction of an aryl urea group appropriately increased the molecular framework and rigidity of ***Compound 7i***, extending it into region B to interact with L85, D86, and R127, which improved its binding affinity with the EGFR. The higher target affinity of ***Compound 7i*** was the main reason for the promising enzyme inhibitory activity. These findings not only verified the scientific design route but also provided valuable clues for the discovery of antitumor compounds based on the EGFR.

## Data Availability

The original contributions presented in the study are included in the article/Supplementary Material, and further inquiries can be directed to the corresponding authors.

## References

[B1] Allam H. A. Aly E. E. Farouk A. K. B. A. W. El Kerdawy A. M. Rashwan E. Abbass S. E. S. (2020). Design and Synthesis of Some New 2,4,6-trisubstituted Quinazoline EGFR Inhibitors as Targeted Anticancer Agents. Bioorg. Chem. 98, 103726. 10.1016/j.bioorg.2020.103726 32171987

[B2] Asquith C. R. M. Maffuid K. A. Laitinen T. Torrice C. D. Tizzard G. J. Crona D. J. (2019). Targeting an EGFR Water Network with 4‐Anilinoquin(az)oline Inhibitors for Chordoma. ChemMedChem 14 (19), 1693–1700. 10.1002/cmdc.201900428 31424613

[B3] Bae J. H. Schlessinger J. (2010). Asymmetric Tyrosine Kinase Arrangements in Activation or Autophosphorylation of Receptor Tyrosine Kinases. Mol. Cell 29 (5), 443–448. 10.1007/s10059-010-0080-5 20432069

[B4] Bazley L. A. Gullick W. J. (2005). The Epidermal Growth Factor Receptor Family. Endocr. Relat. Cancer 12 (Suppl. 1), S17–S27. 10.1677/erc.1.01032 16113093

[B5] Beullens M. Vancauwenbergh S. Morrice N. Derua R. Ceulemans H. Waelkens E. (2005). Substrate Specificity and Activity Regulation of Protein Kinase MELK. J. Biol. Chem. 280 (48), 40003–40011. 10.1074/jbc.m507274200 16216881

[B6] Bishayee S. (2000). Role of Conformational Alteration in the Epidermal Growth Factor Receptor (EGFR) Function. Biochem. Pharmacol. 60 (8), 1217–1223. 10.1016/s0006-2952(00)00425-1 11007960

[B7] Bridges A. J. (2001). Chemical Inhibitors of Protein Kinases. Chem. Rev. 101 (8), 2541–2572. 10.1021/cr000250y 11749388

[B8] David L. Fernandez-Vidal A. Bertoli S. Grgurevic S. Lepage B. Deshaies D. (2016). CHK1 as a Therapeutic Target to Bypass Chemoresistance in AML. Sci. Signal. 9 (445), ra90. 10.1126/scisignal.aac9704 27625304

[B9] Dungo R. T. Keating G. M. (2013). Afatinib: First Global Approval. Drugs 73 (13), 1503–1515. 10.1007/s40265-013-0111-6 23982599

[B10] Eisen T. Joensuu H. Nathan P. Harper P. Wojtukiewicz M. Nicholson S. (2011). 7141 POSTER Phase II Trial of the Oral Multikinase Inhibitor Regorafenib (BAY 73-4506) as First-Line Therapy in Patients with Metastatic or Unresectable Renal Cell Carcinoma (RCC). Eur. J. Cancer 47, S517. 10.1016/s0959-8049(11)72056-1

[B11] El-Sayed M. A.-A. El-Husseiny W. M. Abdel-Aziz N. I. El-Azab A. S. Abuelizz H. A. Abdel-Aziz A. A.-M. (2018). Synthesis and Biological Evaluation of 2-styrylquinolines as Antitumour Agents and EGFR Kinase Inhibitors: Molecular Docking Study. J. Enzyme Inhib. Med. Chem. 33 (1), 199–209. 10.1080/14756366.2017.1407926 29251017 PMC7012010

[B12] Elgazwy A.-S. S. H. Edrees M. M. Ismail N. S. M. (2013). Molecular Modeling Study Bioactive Natural Product of Khellin Analogues as a Novel Potential Pharmacophore of EGFR Inhibitors. J. Enzyme Inhib. Med. Chem. 28 (6), 1171–1181. 10.3109/14756366.2012.719504 23025406

[B13] Fu D.-J. Zhang Y.-F. Chang A.-Q. Li J. (2020). β-Lactams as Promising Anticancer Agents: Molecular Hybrids, Structure Activity Relationships and Potential Targets. Eur. J. Med. Chem. 201, 112510. 10.1016/j.ejmech.2020.112510 32592915

[B14] Garofalo A. Farce A. Ravez S. Lemoine A. Six P. Chavatte P. (2012). Synthesis and Structure-Activity Relationships of (Aryloxy)quinazoline Ureas as Novel, Potent, and Selective Vascular Endothelial Growth Factor Receptor-2 Inhibitors. J. Med. Chem. 55 (3), 1189–1204. 10.1021/jm2013453 22229669

[B15] Gontijo V. S. Viegas F. P. D. Ortiz C. J. C. de Freitas Silva M. Damasio C. M. Rosa M. C. (2020). Molecular Hybridization as a Tool in the Design of Multi-Target Directed Drug Candidates for Neurodegenerative Diseases. Curr. Neuropharmacol. 18 (5), 348–407. 10.2174/1385272823666191021124443 31631821 PMC7457438

[B16] Han Z. He D. Zhang Y. (2020). Genetic Variant Rs7820258 Regulates the Expression of Indoleamine 2,3-dioxygenase 1 in Brain Regions. Proc. Natl. Acad. Sci. U.S.A. 117 (39), 24035–24036. 10.1073/pnas.2007022117 32994208 PMC7533890

[B17] Hirsch F. R. Varella-Garcia M. Bunn P. A. Jr Di Maria M. V. Veve R. Bremnes R. M. (2003). Epidermal Growth Factor Receptor in Non-Small-Cell Lung Carcinomas: Correlation Between Gene Copy Number and Protein Expression and Impact on Prognosis. J. Clin. Oncol. 21 (20), 3798–3807. 10.1200/jco.2003.11.069 12953099

[B18] Huang T. Liu D. Wang Y. Li P. Sun L. Xiong H. (2018). FGFR2 Promotes Gastric Cancer Progression by Inhibiting the Expression of Thrombospondin4 via PI3K-Akt-Mtor Pathway. Cell Physiol. Biochem. 50 (4), 1332–1345. 10.1159/000494590 30355943

[B19] Ju Y. Wu J. Yuan X. Zhao L. Zhang G. Li C. (2018). Design and Evaluation of Potent EGFR Inhibitors through the Incorporation of Macrocyclic Polyamine Moieties Into the 4-Anilinoquinazoline Scaffold. J. Med. Chem. 61 (24), 11372–11383. 10.1021/acs.jmedchem.8b01612 30508379

[B20] Kassouf T. Larive R. Morel A. Urbach S. Bettache N. Marcial Medina M. (2019). The Syk Kinase Promotes Mammary Epithelial Integrity and Inhibits Breast Cancer Invasion by Stabilizing the E-Cadherin/Catenin Complex. Cancers 11 (12), 1974. 10.3390/cancers11121974 PMC6966528 31817924

[B21] Kim H. S. Kim E. Kim J. W. (2001). Development of a Breast Self-Examination Program for the Internet: Health Information for Korean Women. Cancer Nurs. 24 (2), 156–161. 10.1097/00002820-200104000-00012 11318264

[B22] Kim K. W. Roh J. K. Wee H. J. Kim C. (2016). Advancement of the Science and History of Cancer and Anticancer Drugs. Dordrecht, Netherlands: Springer.

[B23] Kobayashi M. Nishita M. Mishima T. Ohashi K. Mizuno K. (2006). MAPKAPK-2-mediated LIM-Kinase Activation is Critical for VEGF-Induced Actin Remodeling and Cell Migration. EMBO J. 25 (4), 713–726. 10.1038/sj.emboj.7600973 16456544 PMC1383554

[B24] Kumar P. Kadyan K. Duhan M. Sindhu J. Singh V. Saharan B. S. (2017). Design, Synthesis, Conformational and Molecular Docking Study of Some Novel Acyl Hydrazone Based Molecular Hybrids as Antimalarial and Antimicrobial Agents. Chem. Cent. J. 11 (1), 115. 10.1186/s13065-017-0344-7 29138944 PMC5686033

[B25] Le Y. Gan Y. Fu Y. Liu J. Li W. Zou X. (2020). Design, Synthesis and *In Vitro* Biological Evaluation of Quinazolinone Derivatives as EGFR Inhibitors for Antitumor Treatment. J. Enzyme Inhib. Med. Chem. 35 (1), 555–564. 10.1080/14756366.2020.1715389 31967481 PMC7006757

[B26] Leick M. B. Levis M. J. (2017). The Future of Targeting FLT3 Activation in AML. Curr. Hematol. Malig Rep. 12 (3), 153–167. 10.1007/s11899-017-0381-2 28421420

[B27] Leroux A. E. Schulze J. O. Biondi R. M. (2018). AGC Kinases, Mechanisms of Regulation and Innovative Drug Development. Semin. Cancer Biol. 48, 1–17. 10.1016/j.semcancer.2017.05.011 28591657

[B28] Liu H. Li F. Zhu Y. Li T. Huang H. Lin T. (2016). Whole-exome Sequencing to Identify Somatic Mutations in Peritoneal Metastatic Gastric Adenocarcinoma: A Preliminary Study. Oncotarget 7 (28), 43894–43906. 10.18632/oncotarget.9707 27270314 PMC5190066

[B29] Malinin N. L. Boldin M. P. Kovalenko A. V. Wallach D. (1997). MAP3K-Related Kinase Involved in NF-kB Induction by TNF, CD95 and IL-1. Nature 385 (6616), 540–544. 10.1038/385540a0 9020361

[B30] Mghwary A. E.-S. Gedawy E. M. Kamal A. M. Abuel-Maaty S. M. (2019). Novel Thienopyrimidine Derivatives as Dual EGFR and VEGFR-2 Inhibitors: Design, Synthesis, Anticancer Activity and Effect on Cell Cycle Profile. J. Enzyme Inhib. Med. Chem. 34 (1), 838–852. 10.1080/14756366.2019.1593160 30919701 PMC6442109

[B31] Mihara M. Shintani S. Nakahara Y. Kiyota A. Ueyama Y. Matsumura T. (2001). Overexpression of CDK2 is a Prognostic Indicator of Oral Cancer Progression. Jpn. J. Cancer Res. 92 (3), 352–360. 10.1111/j.1349-7006.2001.tb01102.x 11267947 PMC5926707

[B32] Mowafy S. Farag N. A. Abouzid K. A. M. (2013). Design, Synthesis and *In Vitro* Anti-Proliferative Activity of 4,6-Quinazolinediamines as Potent EGFR-TK Inhibitors. Eur. J. Med. Chem. 61, 132–145. 10.1016/j.ejmech.2012.10.017 23142066

[B33] Murphy D. A. Makonnen S. Lassoued W. Feldman M. D. Carter C. Lee W. M. F. (2006). Inhibition of Tumor Endothelial ERK Activation, Angiogenesis, and Tumor Growth by Sorafenib (BAY43-9006). Am. J. Pathol. 169 (5), 1875–1885. 10.2353/ajpath.2006.050711 17071608 PMC1780219

[B34] Ogiso H. Ishitani R. Nureki O. Fukai S. Yamanaka M. Kim J.-H. (2002). Crystal Structure of the Complex of Human Epidermal Growth Factor and Receptor Extracellular Domains. Cell 110 (6), 775–787. 10.1016/s0092-8674(02)00963-7 12297050

[B35] Oneyama C. Hikita T. Enya K. Dobenecker M.-W. Saito K. Nada S. (2008). The Lipid Raft-Anchored Adaptor Protein Cbp Controls the Oncogenic Potential of c-Src. Mol. Cel. 30 (4), 426–436. 10.1016/j.molcel.2008.03.026 18498747

[B36] Pflug B. R. Colangelo A. M. Tornatore C. Mocchetti I. (2001). TrkA Induces Differentiation but Not Apoptosis in C6-2B Glioma Cells. J. Neurosci. Res. 64 (6), 636–645. 10.1002/jnr.1117 11398188

[B37] Rao G.-W. Xu G.-J. Wang J. Jiang X.-L. Li H.-B. (2013). Synthesis, Antitumor Evaluation and Docking Study of Novel 4-Anilinoquinazoline Derivatives as Potential Epidermal Growth Factor Receptor (EGFR) Inhibitors. ChemMedChem 8 (6), 928–933. 10.1002/cmdc.201300120 23640754

[B38] Ravez S. Arsenlis S. Barczyk A. Dupont A. Frédérick R. Hesse S. (2015). Synthesis and Biological Evaluation of Di-Aryl Urea Derivatives as C-Kit Inhibitors. Bioorg. Med. Chem. 23 (22), 7340–7347. 10.1016/j.bmc.2015.10.035 26526740

[B39] Reddyrajula R. Dalimba U. Madan Kumar S. (2019). Molecular Hybridization Approach for Phenothiazine Incorporated 1,2,3-Triazole Hybrids as Promising Antimicrobial Agents: Design, Synthesis, Molecular Docking and In Silico ADME Studies. Eur. J. Med. Chem. 168, 263–282. 10.1016/j.ejmech.2019.02.010 30822714

[B40] Sánchez-Bailón M. P. Calcabrini A. Gómez-Domínguez D. Morte B. Martín-Forero E. Gómez-López G. (2012). Src Kinases Catalytic Activity Regulates Proliferation, Migration and Invasiveness of MDA-MB-231 Breast Cancer Cells. Cell Signal. 24 (6), 1276–1286. 10.1016/j.cellsig.2012.02.011 22570868

[B41] Schulze A. Lehmann K. Jefferies H. B. McMahon M. Downward J. (2001). Analysis of the Transcriptional Program Induced by Raf in Epithelial Cells. Genes Dev. 15 (8), 981–994. 10.1101/gad.191101 11316792 PMC312671

[B42] Sever B. Altıntop M. D. Radwan M. O. Özdemir A. Otsuka M. Fujita M. (2019). Design, Synthesis and Biological Evaluation of a New Series of Thiazolyl-Pyrazolines as Dual EGFR and HER2 Inhibitors. Eur. J. Med. Chem. 182, 111648. 10.1016/j.ejmech.2019.111648 31493743

[B43] Simon G. R. Ruckdeschel J. C. Williams C. Cantor A. Chiappori A. Lima C. M. R. (2003). Gefitinib (ZD1839) in Previously Treated Advanced Non-small-cell Lung Cancer: Experience from a Single Institution. Cancer Control 10 (5), 388–395. 10.1177/107327480301000506 14581894

[B44] Smith J. (2005). Erlotinib: Small-Molecule Targeted Therapy in the Treatment of Non-Small-Cell Lung Cancer. Clin. Ther. 27 (10), 1513–1534. 10.1016/j.clinthera.2005.10.014 16330289

[B45] Srivastava R. Geng D. Liu Y. Zheng L. Li Z. Joseph M. A. (2012). Augmentation of Therapeutic Responses in Melanoma by Inhibition of IRAK-1,-4. Cancer Res. 72 (23), 6209–6216. 10.1158/0008-5472.can-12-0337 23041547 PMC3677596

[B46] Stamos J. Sliwkowski M. X. Eigenbrot C. (2002). Structure of the Epidermal Growth Factor Receptor Kinase Domain Alone and in Complex with a 4-Anilinoquinazoline Inhibitor. J. Biol. Chem. 277 (48), 46265–46272. 10.1074/jbc.m207135200 12196540

[B47] Steelman L. S. Pohnert S. C. Shelton J. G. Franklin R. A. Bertrand F. E. McCubrey J. A. (2004). JAK/STAT, Raf/MEK/ERK, PI3K/Akt and BCR-ABL in Cell Cycle Progression and Leukemogenesis. Leukemia 18 (2), 189–218. 10.1038/sj.leu.2403241 14737178

[B48] Sun W.-X. Han H.-W. Yang M.-K. Wen Z.-L. Wang Y.-S. Fu J.-Y. (2019). Design, Synthesis and Biological Evaluation of Benzoylacrylic Acid Shikonin Ester Derivatives as Irreversible Dual Inhibitors of Tubulin and EGFR. Bioorg. Med. Chem. 27 (23), 115153. 10.1016/j.bmc.2019.115153 31648877

[B49] Umekita Y. Ohi Y. Sagara Y. Yoshida H. (2000). Co-Expression of Epidermal Growth Factor Receptor and Transforming Growth Factor-α Predicts Worse Prognosis in Breast-Cancer Patients. Int. J. Cancer 89 (6), 484–487. 10.1002/1097-0215(20001120)89:6<484::aid-ijc3>3.0.co;2-s 11102891

[B50] Vema A. Panigrahi S. K. Rambabu G. Gopalakrishnan B. Sarma J. A. R. P. Desiraju G. R. (2003). Design of EGFR Kinase Inhibitors: A Ligand-Based Approach and its Confirmation with Structure-Based Studies. Bioorg. Med. Chem. 11 (21), 4643–4653. 10.1016/s0968-0896(03)00482-6 14527561

[B51] Wan P. T. C. Garnett M. J. Roe S. M. Lee S. Niculescu-Duvaz D. Good V. M. (2004). Mechanism of Activation of the RAF-ERK Signaling Pathway by Oncogenic Mutations of B-RAF. Cell 116 (6), 855–867. 10.1016/s0092-8674(04)00215-6 15035987

[B52] Wang Z. Wu X. Wang L. Zhang J. Liu J. Song Z. (2016). Facile and Efficient Synthesis and Biological Evaluation of 4-Anilinoquinazoline Derivatives as EGFR Inhibitors. Bioorg. Med. Chem. Lett. 26 (11), 2589–2593. 10.1016/j.bmcl.2016.04.032 27118497

[B53] Wei H. Duan Y. Gou W. Cui J. Ning H. Li D. (2019). Design, Synthesis and Biological Evaluation of Novel 4-Anilinoquinazoline Derivatives as Hypoxia-Selective EGFR and VEGFR-2 Dual Inhibitors. Eur. J. Med. Chem. 181, 111552. 10.1016/j.ejmech.2019.07.055 31387063

[B54] Wilhelm S. Carter C. Lynch M. Lowinger T. Dumas J. Smith R. A. (2006). Discovery and Development of Sorafenib: A Multikinase Inhibitor for Treating Cancer. Nat. Rev. Drug Discov. 5 (10), 835–844. 10.1038/nrd2130 17016424

[B55] Wilhelm S. M. Carter C. Tang L. Wilkie D. McNabola A. Rong H. (2004). BAY 43-9006 Exhibits Broad Spectrum Oral Antitumor Activity and Targets the RAF/MEK/ERK Pathway and Receptor Tyrosine Kinases Involved in Tumor Progression and Angiogenesis. Cancer Res. 64 (19), 7099–7109. 10.1158/0008-5472.can-04-1443 15466206

[B56] Yang Z. Gu J.-M. Ma Q.-Y. Xue N. Shi X.-W. Wang L. (2019). Design, Synthesis and Antitumor Activity of Aromatic Urea-Quinazolines. Future Med. Chem. 11 (21), 2821–2830. 10.4155/fmc-2019-0220 31510797

[B57] Yu L. Huang M. Xu T. Tong L. Yan X.-e. Zhang Z. (2017). A Structure-Guided Optimization of Pyrido[2,3-D]pyrimidin-7-Ones as Selective Inhibitors of EGFRL858R/T790M Mutant with Improved Pharmacokinetic Properties. Eur. J. Med. Chem. 126, 1107–1117. 10.1016/j.ejmech.2016.12.006 28033579

[B58] Zhang H.-Q. Gong F.-H. Ye J.-Q. Zhang C. Yue X.-H. Li C.-G. (2017). Design and Discovery of 4-Anilinoquinazoline-Urea Derivatives as Dual TK Inhibitors of EGFR and VEGFR-2. Eur. J. Med. Chem. 125, 245–254. 10.1016/j.ejmech.2016.09.039 27688180

[B59] Zhang Y. Ying J. B. Hong J. J. Li F. C. Fu T. T. Yang F. Y. (2019). How Does Chirality Determine the Selective Inhibition of Histone Deacetylase 6? A Lesson from Trichostatin A Enantiomers Based on Molecular Dynamics. ACS Chem. Neurosci. 10 (5), 2467–2480. 10.1021/acschemneuro.8b00729 30784262

[B60] Zhang Y. Zheng G. Fu T. Hong J. Li F. Yao X. (2020). The Binding Mode of Vilazodone in the Human Serotonin Transporter Elucidated by Ligand Docking and Molecular Dynamics Simulations. Phys. Chem. Chem. Phys. 22 (9), 5132–5144. 10.1039/c9cp05764a 32073004

[B61] Zhao X. Guan J.-L. (2011). Focal Adhesion Kinase and its Signaling Pathways in Cell Migration and Angiogenesis. Adv. Drug Deliv. Rev. 63 (8), 610–615. 10.1016/j.addr.2010.11.001 21118706 PMC3132829

[B62] Zheng Y.-G. Su J. Gao C.-Y. Jiang P. An L. Xue Y.-S. (2017). Design, Synthesis, and Biological Evaluation of Novel 4-Anilinoquinazoline Derivatives Bearing Amino Acid Moiety as Potential EGFR Kinase Inhibitors. Eur. J. Med. Chem. 130, 393–405. 10.1016/j.ejmech.2017.02.061 28279846

[B63] Zhu H. Tang L. Zhang C. Wei B. Yang P. He D. (2019). Synthesis of Chalcone Derivatives: Inducing Apoptosis of HepG2 Cells via Regulating Reactive Oxygen Species and Mitochondrial Pathway. Front. Pharmacol. 10, 1341. 10.3389/fphar.2019.01341 31803052 PMC6874057

