# Supplementary material for: Discovery of Potent EGFR Inhibitors With 6-Arylureido-4-anilinoquinazoline Derivatives
Source: Front Pharmacol. 2021 May 26;12:647591. doi: 10.3389/fphar.2021.647591 (PMC8187944; doi:10.3389/fphar.2021.647591)
Supplement: Supplementary file 1 [file DataSheet1.docx]

**Supplementary Information:**

**Discovery of Potent EGFR Inhibitors with *6-arylureido-4-anilinoquinazoline* Derivatives**

Meng Li^1,§^, Na Xue^2,§^, Xingang Liu^1^, Qiaoyun Wang^1^, Hongyi Yan^1^, Yifan Liu^1^, Lei Wang^1^, Xiaowei Shi^1^, Deying Cao^1^, Kai Zhang^*,1^ and Yang Zhang^*,1^

^1^Department of Medicinal Chemistry, Hebei Medical University, Shijiazhuang 050017, China.

^2^Department of Pharmaceutical Engineering, Hebei Chemical and Pharmaceutical College, 050026, Shijiazhuang, China.

**Corresponding Authors**

*Kai Zhang (zhk810728@163.com), Department of Medicinal Chemistry, Hebei Medical University, Shijiazhuang 050017, China.

*Yang Zhang (zhangyang_16@hebmu.edu.cn), Department of Medicinal Chemistry, Hebei Medical University, Shijiazhuang 050017, China.

^§^M. L. and N. X. contributed equally to this study.

Synthesis and characterization of the target compounds

^1^H NMR and ^13^C NMR spectra were recorded in DMSO-*d_6_* solution using a Bruker AV-400 spectrometer. All chemical shifts were reported in ppm (δ). Infrared (IR) spectra were recorded on a SHIMADZU FTIR-8400S spectrometer (KBr disks). Mass spectrometry was obtained using a 3200 QTRAP and a Triple Q-tof 5600+ high resolution mass spectrometer (AB/SCIEX). Melting points were determined on a M-560 MP apparatus (BUCHI) and were uncorrected. All reactions were monitored by thin-layer chromatography (TLC) in silica gel (purchased from Yantai Xinnuo Chemical Plant) and the products were visualized with ultraviolet lamp (254 and 365 nm).

*6-nitroquinazolin-4(3H)-one* ***(1)***

A mixture of 2-amino-4-nitrobenzoic acid (7.28 g, 40.0 mmol) and formamide (60 mL) was stirred at 150 °C for 16 h and monitored with TLC. Then the mixture was cooled to room temperature. Then filtered and the filter cake was washed with 2-propanol and dried to give compound 2. Yield: 51.6%; m.p. 285.0-286.0 °C; ^1^H NMR (400 MHz-DMSO-*d_6_*): δ 12.809 (brs, 1H), 8.791-8.784(d, *J* =2.8 Hz, 1H), 8.551-8.529 (dd, *J* = 2.4 Hz, 8.8 Hz, 1H), 8.314 (s, 1H), 7.866-7.843 (d, *J* = 9.2 Hz, 1H), MS (ESI^-^) m/z 190.0 (M-H)^-^.

*4-chloro-6-nitroquinazoline* ***(2)***

A suspension of compound 1 (2.25 g, 11.8 mmol), thionyl chloride (23 mL), DMF (0.2 mL) was raised to reflux for 2.5 h until the solution clear. After that, the solution was cooled to room temperature, and the solvent was removed under reduced pressure, the residue was diluted with CH_2_Cl_2_ and concentrated again to provide compound 2 as yellow solid, which was used for next step without further purification. m.p. 130 ℃; ^1^H NMR (400 MHz-DMSO-*d_6_*): δ 8.78 (d, *J* = 2 Hz, 1H),8.555 (dd, *J*= 6.7 Hz, 2 Hz, 1H), 8.432 (1H, s), 7.833 (d, *J* = 6.7 Hz, 1H). MS (ESI^-^) m/z 208.2 (M-H)^-^.

*Synthesis of* ***compounds 3a-i***

To the solution of compound 2 (2.65 g, 11.8 mmol) in 2-propanol (37 mL), corresponding aniline (14.1 mmol) was added at room temperature, and then the mixture was reflux. Once the reaction was completed as indicated by TLC, the mixture was cooled to room temperature and the resultant precipitate was collected by filtration and washed with 2-propanol.

*N-(3-bromophenyl)-6-nitroquinazolin-4-amine* ***(3a)***

Yield: 85.2%; m.p. 287.8~289.6 °C; ^1^H NMR (400 MHz-DMSO-*d_6_*): δ 12.088- 12.067 (brs, 1H), 9.921-9.917 (d, *J* = 1.6 Hz, 1H), 8.999 (s, 1H), 8.783-8.755 (dd, *J* = 2.0 Hz, 9.2 Hz, 1H), 8.200-8.177 (d, *J* = 9.2 Hz, 1H), 7.778-7.285 (m, 4H). MS (ESI^+^) m/z 347.2 (M+H)^+^.

*N-(4-methoxyphenyl)-6-nitroquinazolin-4-amine* ***(3b)***

Yield: 88.2%; m.p. 274.2~276.6 °C; ^1^H NMR (400 MHz-DMSO-*d_6_*): δ 12.180 (brs, 1H), 9.913-9.908 (d, *J* = 2.0 Hz, 1H), 8.960 (s, 1H), 8.788-8.760 (dd, *J* = 2.0 Hz, 9.2 Hz, 1H), 8.205-8.182 (d, *J* = 9.2 Hz, 1H), 7.668-7.646 (d, *J* = 8.8 Hz, 2H), 7.077-7.054 (d, *J* = 9.2 Hz, 2H), 3.815-3.760 (m, 3H), MS (ESI^+^) m/z 297.2 (M+H)^+^.

*N-(4-bromo-2-fluorophenyl)-6-nitroquinazolin-4-amine* ***(3c)***

Yield: 72.0%; m.p. 291.4~292.7 °C; ^1^H NMR (400 MHz-DMSO-*d_6_*): δ 9.902 (s, 1H), 8.989 (s, 1H), 8.818-8.790 (dd, *J* = 2.0 Hz, 9.2 Hz, 1H), 8.239-8.216 (d, *J* = 9.2 Hz, 1H), 7.821-7.796 (d, *J* = 6.4 Hz, 1H), 7.612-7.567 (m, 2H), MS (ESI^+^) m/z 365.1 (M+H)^+^.

*N-(3-chloro-4-fluorophenyl)-6-nitroquinazolin-4-amine* ***(3d)***

Yield: 76.3%; m.p. 289.4~290.5 °C; ^1^H NMR (400 MHz-DMSO-*d_6_*): δ 12.432-12.401 (brs, 1H), 9.982-9.978 (d, *J* = 1.6 Hz, 1H), 9.060 (s, 1H), 8.790-8.761 (dd, *J* = 2.4 Hz, 9.2 Hz, 1H), 8.230-8.207 (d, *J* = 9.2 Hz, 1H), 8.079-8.056 (m, 1H), 7.822-7.783 (m, 1H), 7.591-7.545 (m, 1H). MS (ESI^+^) m/z 319.2 (M+H)^+^.

*N-mesityl-6-nitroquinazolin-4-amine* ***(3e)***

Yield: 80.2%; m.p. 261.4~262.0 °C; ^1^H NMR (400 MHz-DMSO-*d_6_*): δ 9.966 (s, 1H), 8.868-8.792 (m, 1H), 8.240-8.217 (d, *J* = 9.2 Hz, 1H), 7.024-6.943 (m, 4H), 2.224 (s, 6H), 2.137 (s, 3H). MS (ESI^+^) m/z 309.3 (M+H)^+^.

*N-(4-fluorophenyl)-6-nitroquinazolin-4-amine* ***(3f)***

Yield: 97.2%; m.p. 234.1~236.4 °C; ^1^H NMR (400 MHz-DMSO-*d_6_*): δ 10.736 (brs, 1H), 9.654-9.649 (d, *J* = 2.0 Hz, 1H), 8.728 (s, 1H), 8.586-8.558 (dd, *J* = 2.0 Hz, 9.2 Hz, 1H), 7.951-7.928 (d, *J* = 9.2 Hz, 1H), 7.845-7.810 (m, 2H), 7.309-7.265 (m, 2H). MS (ESI^+^) m/z 285.2 (M+H)^+^.

*6-nitro-N-(p-tolyl)quinazolin-4-amine* ***(3g)***

Yield: 90.5%; m.p. 280.3~282.1 °C; ^1^H NMR (400 MHz-DMSO-*d_6_*): δ 12.108(s, 1H), 9.915-9.910 (d, *J* = 2.0 Hz, 1H), 8.966 (s, 1H), 8.787-8.758 (dd, *J* = 2.4 Hz, 9.2 Hz, 1H), 8.203-8.180 (d, *J* = 9.2 Hz, 1H), 7.614-7.635 (d, *J* = 8.4 Hz , 2H), 7.321-7.295 (m , 2H),2.362(s,3H). MS (ESI^+^) m/z 281.2 (M+H)^+^.

*N-(2,3-dimethylphenyl)-6-nitroquinazolin-4-amine* ***(3h)***

Yield: 98.5%; m.p. 280.9~283.2 °C; ^1^H NMR (400 MHz-DMSO-*d_6_*): δ 12.308 (brs, 1H), 9.915 (s, 1H), 8.886 (s, 1H), 8.817-8.794 (d, *J* = 9.2 Hz, 1H), 8.225-8.202 (d, *J* = 9.2 Hz, 1H), 7.266-7.182 (m, 3H), 2.334 (s, 3H), 2.119 (s, 3H). MS (ESI^+^) m/z 295.1 (M+H)^+^.

*N-(4-bromophenyl)-6-nitroquinazolin-4-amine* ***(3i)***

Yield: 92.4%; m.p. 306.2~308.7 °C; ^1^H NMR (400 MHz-DMSO-*d_6_*): δ 12.115-12.091 (brs, 1H), 9.932-9.927 (d, *J* = 2.0 Hz, 1H), 9.039 (s, 1H), 8.786-8.758 (dd, *J* = 2.0 Hz, 9.2 Hz, 1H), 8.205-8.182 (d, *J* = 9.2 Hz, 1H), 8.080-8.072 (t, *J* = 1.6 Hz, 1H), 7.830-7.810 (d, *J* = 8.0 Hz, 1H), 7.548-7.456 (m, 2H). MS (ESI^-^) m/z 343.0 (M-H)^-^.

*Synthesis of* ***compounds 4a-i***

A mixture of 3a-i (4.90 mmol) and stannous chloride (19.6 mmol) in ethyl acetate (49 mL) was stirred for 1h at reflux. After the mixture was cooled to room temperature, the solid precipitate was filtered under vacuum through celite and washed with ethyl acetate (100 mL×3). The aqueous layer of resulting mixture was neutralized with saturated Na_2_CO_3_ to pH 7. The organic phase was separated from the aqueous phase and abandoned. The aqueous phase was extracted with ethyl acetate (2×50 mL), the organic fractions were combined, washed with water (100 mL×3), dried (Na_2_SO_4_), filtered, and concentrated under reduced pressure.

*N^4^-(3-bromophenyl) quinazoline-4,6-diamine* ***(4a)***

Yield: 55.1%; m.p. 191.7~193.6 °C; ^1^H NMR (400 MHz-DMSO-*d_6_*): δ 9.455 (s, 1H), 8.368 (s, 1H), 7.955-7.810 (m, 2H), 7.707-7.527 (m, 3H), 7.366-7.362 (d, *J* = 1.6 Hz, 1H), 7.287-7.265 (d, *J* = 8.8 Hz, 1H), 5.629 (s, 2H). MS (ESI^+^) m/z 316.3 (M+H)^+^.

*N^4^-(4-methoxyphenyl) quinazoline-4,6-diamine* ***(4b)***

Yield: 65.2%; m.p. 130.1~132.5 °C; ^1^H NMR (400 MHz-DMSO-*d_6_*): δ 9.282 (s, 1H), 8.302 (s, 1H), 7.751-7.729 (d, *J* = 8.8 Hz, 2H), 7.560-7.538 (d, *J* = 8.8 Hz, 1H), 7.384-7.380 (d, *J* = 1.6 Hz, 1H), 7.279-7.252 (dd, *J* = 2.0 Hz, 8.8 Hz, 1H), 6.995-6.973 (d, *J* = 8.8 Hz, 2H), 5.571 (s, 2H), 3.802 (s, 3H). MS (ESI^+^) m/z 267.1 (M+H)^+^.

*N^4^-(4-bromo-2-fluorophenyl) quinazoline-4,6-diamine* ***(4c)***

Yield: 75.3%; m.p. 197.6~199.2 °C; ^1^H NMR (400 MHz-DMSO-*d_6_*): δ 9.319 (s, 1H), 8.228 (s, 1H), 7.636-7.433 (m, 4H), 7.265-7.244 (d, *J* = 8.4 Hz, 2H), 5.649 (s, 2H). MS (ESI^-^) m/z 333.0 (M**-**H)^-^.

*N^4^-(3-chloro-4-fluorophenyl) quinazoline-4,6-diamine* ***(4d)***

Yield: 68.4%; m.p. 247.8~249.9 °C; ^1^H NMR (400 MHz-DMSO-*d_6_*): δ 9.498 (s, 1H), 8.389 (s, 1H), 8.242-8.219 (dd, *J* = 2.4 Hz, 6.8 Hz,1H), 7.863-7.830 (m, 1H), 7.584-7.562 (d, *J* = 8.8 Hz, 1H), 7.441-7.270 (m, 3H), 5.654 (s, 2H). MS (ESI^+^) m/z 291.0 (M+H)^+^.

*N^4^-mesitylquinazoline-4,6-diamine* ***(4e)***

Yield: 32.4%; m.p. 268.8~270.1 °C; ^1^H NMR (400 MHz-DMSO-*d_6_*): δ 8.916 (s, 1H), 8.050 (s, 1H), 7.494-7.472 (d, *J* = 8.8 Hz, 1H), 7.316-7.311 (d, *J* = 2.0 Hz, 1H), 7.223-7.194 (dd, *J* = 2.4 Hz, 8.8Hz, 1H), 6.938 (s, 2H), 5.486 (s, 2H), 2.273 (s, 3H), 2.038 (s, 6H), MS (ESI^+^) m/z 279.3 (M+H)^+^.

*N^4^-(4-fluorophenyl) quinazoline-4,6-diamine* ***(4f)***

Yield: 74.3%; m.p. 173.0~174.6 °C; ^1^H NMR (400 MHz-DMSO-*d_6_*): δ 9.396 (s, 1H), 8.318 (s, 1H), 7.862 (s, 2H), 7.553-7.532 (d, *J* = 8.4 Hz, 1H), 7.353 (s, 1H), 7.270-7.182 (m, 3H), 5.597 (s, 2H). MS (ESI^+^) m/z 255.2 (M+H)^+^.

*N^4^-(p-tolyl) quinazoline-4,6-diamine* ***(4g)***

Yield: 65.5%; m.p. 178.7~180.0 °C; ^1^H NMR (400 MHz-DMSO-*d_6_*): δ 9.258 (s, 1H), 8.293 (s, 1H), 7.732-7.712 (d, *J* = 8.8 Hz, 2H), 7.527-7.505 (d, *J* = 8.8 Hz, 1H), 7.358-7.353 (d, *J* = 1.6 Hz, 1H), 7.245-7.150 (m, 3H), 5.544 (s, 2H),2.295(s, 3H). MS (ESI^+^) m/z 251.2 (M+H)^+^.

*N^4^-(2, 3-dimethylphenyl) quinazoline-4,6-diamine* ***(4h)***

Yield: 56.2%; m.p. 178.9~181.2 °C; ^1^H NMR (400 MHz-DMSO-*d_6_*): δ 9.170 (s, 1H), 8.103 (s, 1H), 7.502-7.480 (d, *J* = 8.8 Hz, 1H), 7.301 (s, 1H), 7.222-7.200 (d, *J* = 8.8 Hz, 1H), 7.108-7.095 (d, *J* = 5.2 Hz, 3H), 5.516 (s, 2H), 2.290 (s, 3H), 2.034 (s, 3H).MS (ESI^+^) m/z 265.3 (M+H)^+^.

*N^4^-(4-bromophenyl) quinazoline-4,6-diamine* ***(4i)***

Yield: 57.3%; m.p. 211.0~312.6 °C; ^1^H NMR (400 MHz-DMSO-*d_6_*): δ 9.467 (s, 1H), 8.402 (s, 1H), 8.254 (s, 1H), 7.913-7.893 (d, *J* = 8.0 Hz, 1H), 7.582-7.560 (d, *J* = 8.8 Hz, 1H), 7.363-7.231 (m, 4H), 5.646 (s, 2H), MS (ESI^+^) m/z 315.2 (M+H)^+^.

*Synthesis of* ***compounds 6a-d***

To a stirred solution of triphosgene (43 mmol) in dichloromethane (200 mL) was added corresponding aniline (5a-d) (109 mmol) and triethylamine (224 mmol) dropwise at 0-5 ℃, then refluxed for 3 h, cooled to room temperature and concentrated. The residual was further purified by vacuum distillation. Because of the toxicity of isocyanate, input it to the next reaction as soon as possible.

*Synthesis of* ***compounds 7a-t***

To the solution of 6a-d (2.2 mmol) and anhydrous acetonitrile (3 mL), compound 4a-i (2.0 mmol) was added at room temperature. After 3 h, the product was filtered off, and the solid was washed with anhydrous acetonitrile and dried.

*1-(4-((3-bromophenyl) amino)-3,4-dihydroquinazolin-6-yl)-3-phenylurea* ***(7a)***

Yield: 78.5%; m.p.250.0-250.9 °C; compound purity: 96.714 %; aqueous solubility: soluble; IR (KBr) γ/cm^-1^: 3327, 3202, 3121, 3090, 3034, 1701, 1614, 1599, 1566, 1524, 1499, 1475, 1445, 1410, 1364, 1317, 1277, 1217, 835, 771, 752, 679;^1^H NMR (400 MHz-DMSO-*d_6_*): δ 9.856 (s, 1H), 8.979-8.897 (d, *J* = 32.8 Hz, 2H), 8.564-8.505 (d, *J* = 23.6 Hz, 2H), 8.201 (s, 1H), 7.891-7.790 (m, 3H), 7.524 (s, 2H), 7.311 (s, 4H), 7.013-7.001 (d, *J* = 4.8Hz, 1H); ^13^C NMR (100 MHz-DMSO-*d_6_*): δ 156.97, 152.56, 152.45, 145.85, 141.23, 139.49, 137.72, 130.29, 128.82, 128.50, 126.58, 125.73, 124.15, 122.05, 121.11, 120.75, 118.31, 115.62,110.00; HRMS (ESI): m/z calculated for (C_21_H_16_BrN_5_O+H)^+^: 434.0616; found: 434.0611.

1-(4-((4-methoxyphenyl) amino)-3,4-dihydroquinazolin-6-yl)-3-phenylurea (***7b***)

Yield: 57.4%; m.p. 209.6-210.2 ℃; compound purity: 97.683 %; aqueous solubility: soluble; IR (KBr) γ/cm^-1^: 3288, 3248, 2833, 2750, 1718, 1610, 1599, 1574, 1523, 1508, 1441, 1306, 1250, 1219, 1194, 1028, 829, 750, 689; ^1^H NMR (400 MHz-DMSO-*d_6_*): δ 10.542 (brs, 1H), 9.344 (s, 1H), 9.219 (s, 1H), 8.595 (s, 2H), 7.988-7.961 (dd, *J* = 1.6 Hz, 9.2 Hz, 1H), 7.792-7.770 (d, *J* = 8.8 Hz, 1H), 7.629-7.607 (d, *J* = 8.8 Hz, 2H), 7.527-7.507 (d, *J* = 8.0 Hz, 2H), 7.327-7.288 (t, *J* = 8.0 Hz, 2H), 7.019-6.981 (m, 3H), 3.658 (s, 3H); ^13^C NMR (100 MHz-DMSO-*d_6_*): δ 158.30, 156.72, 152.58, 150.77, 139.53, 139.30, 138.81, 130.81, 128.81, 127.28, 125.42, 124.16, 122.01, 118.16, 114.77, 113.73, 110.34, 55.26; HRMS (ESI): m/z calculated for (C_22_H_19_N_5_O_2_+H)^+^: 386.1617; found: 386.1617.

1-(4-((4-bromo-2-fluorophenyl) amino)-3,4-dihydroquinazolin-6-yl)-3-phenylurea (***7c***)

Yield: 65.7%; m.p. 256.0-256.8 ℃; compound purity: 98.727 %; aqueous solubility: soluble; IR (KBr) γ/cm^-1^: 3310, 3049, 1717, 1697, 1683, 1595, 1576, 1529, 1497, 1443, 1410, 1312, 1225, 1190, 874, 837, 746, 690 cm^-1^; ^1^H NMR (400 MHz-DMSO-*d_6_*): δ 9.790 (s, 1H), 9.000 (s, 1H), 8.869 (s, 1H), 8.491-8.487 (d, *J* = 1.6 Hz, 1H), 8.405 (s, 1H), 7.862-7.754 (m, 1H), 7.668-7.663 (d, *J* = 2.0 Hz, 1H), 7.643-7.638 (d, *J* = 2.0 Hz, 1H), 7.557-7.459 (m, 4H), 7.327-7.287 (t, *J* = 8.0 Hz, 2H), 7.016-6.980 (t, *J* = 7.2 Hz, 1H); ^13^C NMR (100 MHz-DMSO-*d_6_*): δ 157.78, 152.70, 152.52, 145.65, 139.48, 137.69, 129.33, 128.81, 128.43, 127.47, 126.49, 126.40, 122.05, 119.36, 119.13, 118.30, 115.24, 109.67; HRMS (ESI): m/z calculated for (C_21_H_15_BrFN_5_O+H)^+^: 452.0522; found: 452.0519.

1-(4-((3-chloro-4-fluorophenyl) amino)-3,4-dihydroquinazolin-6-yl)-3-phenylurea (***7d***)

Yield: 45.0%; m.p. 233.0-233.7 ℃; compound purity: 99.153 %; aqueous solubility: soluble; IR (KBr) γ/cm^-1^: 3283, 3067, 3032, 1697, 1618, 1599, 1576, 1555, 1501, 1445, 1427, 1387, 1364, 1312, 1211, 933, 876, 837, 777, 746, 733, 689 cm^-1^; ^1^H NMR (400 MHz-DMSO-*d_6_*): δ 9.884 (s, 1H), 8.984 (s, 1H), 8.893 (s, 1H), 8.545 (s, 1H), 8.504 (s, 1H), 8.180-8.163 (m, 1H), 7.894-7.766 (m, 3H), 7.530-7.422 (m, 3H), 7.332-7.293 (m, 2H), 7.021-6.987 (m, 1H); ^13^C NMR (100 MHz-DMSO-*d_6_*): δ 157.50, 154.91, 153.05, 152.94, 152.50, 146.28, 139.99, 138.22, 137.26, 129.32, 129.00, 127.04, 124.13, 123.07, 123.00, 122.55, 119.27, 119.09, 118.81, 117.06, 116.85, 115.99, 110.38; HRMS (ESI): m/z calculated for (C_21_H_15_ClFN_5_O+H)^+^: 408.1027; found: 408.1046.

1-(4-(mesitylamino)-3,4-dihydroquinazolin-6-yl)-3-phenylurea (***7e***)

Yield: 66.7%; m.p. 169.4-171.4 ℃; compound purity: 99.429 %; aqueous solubility: soluble; IR (KBr) γ/cm^-1^: 3308, 3209, 2918, 1697, 1685, 1578, 1531, 1499, 1441, 1412, 1373, 1313, 1277, 1219, 1026, 837, 750, 690 cm^-1^; ^1^H NMR (400 MHz-DMSO-*d_6_*): δ 9.365 (s, 1H), 8.895 (s, 1H), 8.854 (s, 1H), 8.383 (s, 1H), 8.232 (s, 1H), 7.921-7.901 (d, *J* = 8.0 Hz, 1H), 7.714-7.692 (d, *J* = 8.8 Hz, 1H), 7.516-7.496 (d, *J* = 8.0 Hz, 2H), 7.319-7.301 (d, *J* = 7.2 Hz, 2H), 6.991-6.958 (m, 3H), 2.283 (s, 3H), 2.090 (s, 6H); ^13^C NMR (100 MHz-DMSO-*d_6_*): δ 158.86, 153.94, 153.06, 146.04, 140.05, 137.70, 136.09, 136.01, 134.19, 129.29, 128.90, 128.75, 126.63, 122.47, 118.77, 115.42, 110.77, 21.06, 18.46; HRMS (ESI): m/z calculated for (C_24_H_23_N_5_O+ H)^+^: 398.1980; found: 398.1990.

1-(4-((4-fluorophenyl) amino)-3,4-dihydroquinazolin-6-yl)-3-phenylurea (***7f***)

Yield: 68.3%; m.p. 252.8-253.5 ℃; compound purity: 99.161 %; aqueous solubility: soluble; IR (KBr) γ/cm^-1^: 3300, 3142, 3034, 1703, 1578, 1545, 1508, 1425, 1366, 1313, 1223, 1159, 831, 746, 690 cm^-1^; ^1^H NMR (400 MHz-DMSO-*d_6_*): δ 9.782 (s, 1H), 8.938 (s, 1H), 8.881(s, 1H), 8.469 (s, 2H), 7.895-7.735 (m, 4H), 7.521-7.500 (d, *J* = 8.4 Hz, 2H), 7.326-7.286 (t, *J* = 8.0 Hz, 2H), 7.247-7.203 (t, *J* = 8.8 Hz, 2H), 7.015-6.979 (t, *J* = 7.2 Hz, 1H); ^13^C NMR (100 MHz-DMSO-*d_6_*): δ 152.68, 152.57, 139.52, 137.52, 128.81, 128.39, 126.38, 124.44, 124.36, 122.03, 118.29, 115.48, 115.05, 114.83, 110.13; HRMS (ESI): m/z calculated for (C_21_H_16_FN_5_O+H)^+^: 374.1417; found: 374.1414.

1-phenyl-3-(4-(p-tolylamino)-3,4-dihydroquinazolin-6-yl) urea (***7g***)

Yield: 75.0%; m.p. 252.2-253.0 ℃; compound purity: 99.444 %; aqueous solubility: soluble; IR (KBr) γ/cm^-1^: 3319, 3290, 3217, 3030, 1705, 1632, 1618, 1599, 1533, 1514, 1501, 1433, 1364, 1313, 1219, 928, 887, 831, 816, 804, 746, 688, 669 cm^-1^; ^1^H NMR (400 MHz-DMSO-*d_6_*): δ 9.678 (s, 1H), 8.920 (s, 1H), 8.890 (s, 1H), 8.458-8.449 (d, *J* = 3.6 Hz, 2H), 7.909-7.882 (dd, *J* = 2.0 Hz, 8.8 Hz, 1H), 7.747-7.724 (d, *J* = 9.2 Hz, 1H), 7.702-7.681 (d, *J* = 8.4 Hz, 2H), 7.524-7.504 (d, *J* = 8.0 Hz, 2H), 7.328-7.289 (t, *J* = 8.0 Hz, 2H), 7.203-7.183 (d, *J* = 8.0Hz, 2H), 7.016-6.980 (t, *J* = 7.2 Hz, 1H), 2.313 (s, 3H); ^13^C NMR (100 MHz-DMSO-*d_6_*): δ 157.29, 152.81, 152.59, 145.73, 139.52, 137.40, 136.78, 132.52, 128.81, 128.79, 128.32, 126.30, 122.52, 122.02, 118.29, 115.56, 110.30, 20.51; HRMS (ESI): m/z calculated for (C_22_H_19_N_5_O+H)^+^: 370.1667; found: 370.1670.

1-(4-((2,3-dimethylphenyl) amino)-3,4-dihydroquinazolin-6-yl)-3-phenylurea (***7h***)

Yield: 34.0%; m.p. 242.8-243.7 ℃; compound purity: 99.169 %; aqueous solubility: soluble; IR (KBr) γ/cm^-1^: 3329, 3140, 3032, 1709, 1614, 1578, 1545, 1599, 1474, 1445, 1431, 1418, 1367, 1339, 1313, 1225, 930, 829, 775, 743, 687 cm^-1^; ^1^H NMR (400 MHz-DMSO-*d_6_*): δ 9.639 (s, 1H), 8.914 (s, 1H), 8.871 (s, 1H), 8.417 (s, 1H), 8.287 (s, 1H), 7.913-7.891 (d, *J* = 8.8 Hz, 1H), 7.726-7.704 (d, *J* = 8.8 Hz, 1H), 7.521- 7.501 (d, *J* = 8.0 Hz, 2H), 7.322-7.284 (t, *J* = 7.6 Hz, 2H), 7.130 (s, 3H), 7.010 -6.974 (t, *J* = 7.2 Hz, 1H), 2.304 (s, 3H), 2.050 (s, 3H); ^13^C NMR (100 MHz-DMSO-*d_6_*): δ 159.05, 153.70, 153.06, 146.07, 140.05, 137.91, 137.79, 137.53, 134.16, 129.30, 128.72, 128.12, 126.61, 125.91, 122.48, 118.77, 115.64, 110.75, 20.63, 14.88; HRMS (ESI): m/z calculated for (C_23_H_21_N_5_O+H)^+^: 384.1824; found: 384.1832.

1-(4-chloro-3-(trifluoromethyl) phenyl)-3-(4-((4-methoxyphenyl) amino)-3,4-dihydroquinazolin-6-yl) urea (***7j***)

Yield: 82.3%; m.p. 255.4-255.9 ℃; compound purity: 98.515 %; aqueous solubility: soluble; IR (KBr) γ/cm^-1^: 3325, 3003, 2932, 2835, 1711, 1607, 1576, 1541, 1508, 1481, 1421, 1364, 1327, 1254, 1215, 1175, 1124, 1030, 827, 663 cm^-1^; ^1^H NMR (400 MHz-DMSO-*d_6_*): δ 9.702 (s, 1H), 9.384 (s, 1H), 9.100 (s, 1H), 8.487-8.482 (d, *J* = 2.0 Hz, 1H), 8.434 (s, 1H), 8.204- 8.198 (d, *J* = 2.4 Hz, 1H), 7.861-7.833 (dd, *J* = 2.0 Hz, 9.2 Hz, 1H), 7.743-7.632 (m, 5H), 6.982-6.959 (d, *J* = 9.2 Hz, 2H), 3.778 (s, 3H); ^13^C NMR (100 MHz-DMSO-*d_6_*): δ 157.49, 155.77, 153.07, 152.51, 145.75, 139.25, 136.78, 132.03, 128.20, 126.87, 126.56. 124.52, 124.15, 123.14, 122.47, 121.44, 116.84, 116.78, 115.37, 113.57, 111.09, 55.18; HRMS (ESI): m/z calculated for (C_23_H_17_ClF_3_N_5_O_2_+H)^+^: 488.1101; found: 488.1089.

1-(4-((4-bromophenyl) amino)-3,4-dihydroquinazolin-6-yl)-3-(4-chloro-3-(trifluoromethyl) phenyl) urea (***7k***)

Yield: 86.8%; m.p. 269.8-271.1 ℃; compound purity: 99.781 %; aqueous solubility: soluble; IR (KBr) γ/cm^-1^: 3580, 3335, 3211, 3140, 3057, 1699, 1616, 1599, 1553, 1489, 1423, 1327, 1286, 1263, 1221, 1194, 1130, 901, 816, 679 cm^-1^; ^1^H NMR (400 MHz-DMSO-*d_6_*): δ 9.850 (s, 1H), 9.377 (s, 1H), 9.143 (s, 1H), 8.533 (s, 2H), 8.198- 8.192 (d, *J* = 2.4 Hz, 1H), 7.885-7.835 (m, 3H), 7.790-7.768 (d, *J* = 2.8 Hz, 1H), 7.708-7.634 (m, 2H), 7.578-7.556 (d, *J* = 8.8 Hz, 2H); ^13^C NMR (100 MHz-DMSO-*d_6_*): δ 157.07, 152.74, 152.50, 146.08, 139.20, 138.83, 137.06, 132.04, 131.14, 128.49, 126.81, 124.16, 123.17, 122.52, 116.88, 116.82, 115.55, 115.09, 110.86; HRMS (ESI): m/z calculated for (C_22_H_14_BrClF_3_N_5_O+H)^+^: 536.0100; found: 536.0087.

1-(4-chloro-3-(trifluoromethyl) phenyl)-3-(4-((4-fluorophenyl) amino)-3,4-dihydroquinazolin-6-yl) urea (***7l***)

Yield: 82.0%; m.p. 246.8-247.9 ℃; compound purity: 99.689 %; aqueous solubility: soluble; IR (KBr) γ/cm^-1^: 3649, 3339, 3074, 1705, 1582, 1556, 1508, 1485, 1435, 1327, 1223, 1130, 1032, 833, 808 cm^-1^; ^1^H NMR (400 MHz-DMSO-*d_6_*): δ 9.808 (s, 1H), 9.373 (s, 1H), 9.126 (s, 1H), 8.516-8.485 (d, *J* = 12.4 Hz, 2H), 8.200 (s, 1H), 7.868-7.752 (m, 4H), 7.700-7.634 (m, 2H), 7.252-7.208 (t, *J* = 8.8 Hz, 2H); ^13^C NMR (400 MHz-DMSO-*d_6_*): δ 157.35, 152.92, 152.50, 146.00, 139.22, 136.94, 135.60, 132.04, 128.43, 126.87, 126.70, 124.59, 124.51, 123.16, 116.86, 116.81, 115.40, 115.05, 114.83, 110.91; HRMS (ESI): m/z calculated for (C_22_H_14_ClF_4_N_5_O+H)^+^: 476.0901; found: 476.0086.

1-(4-chloro-3-(trifluoromethyl) phenyl)-3-(4-(p-tolylamino)-3,4-dihydroquina-zolin-6-yl) urea (***7m***)

Yield: 78.2%; m.p. 256.8-258.3 ℃; compound purity: 97.586 %; aqueous solubility: soluble; IR (KBr) γ/cm^-1^: 3327, 3275, 2920, 1709, 1616, 1541, 1526, 1508, 1481, 1420, 1327, 1217, 1177, 831, 665 cm^-1^; ^1^H NMR (400 MHz-DMSO-*d_6_*): δ 9.734 (s, 1H), 9.406 (s, 1H), 9.123 (s, 1H), 8.507-8.502 (d, *J* = 2.0 Hz, 1H), 8.473 (s, 1H), 8.201-8.195 (d, *J* = 2.4 Hz, 1H), 7.878-7.850 (dd, *J* = 2.0 Hz, 9.2 Hz, 1H), 7.757-7.735 (d, *J* = 8.8 Hz, 1H), 7.704-7.632 (m, 4H), 7.207-7.186 (d, *J* = 8.4 Hz, 2H), 2.316 (s, 3H); ^13^C NMR (100 MHz-DMSO-*d_6_*): δ 157.39, 152.96, 152.52, 145.76, 139.24, 136.87, 136.67, 132.67, 132.03, 128.79, 128.21, 126.87, 126,65, 126,57, 124.15, 123.13, 122.69, 122.48, 121.44, 116.84, 116.78, 115.47, 111.11, 20.51; HRMS (ESI): m/z calculated for (C_23_H_17_ClF_3_N_5_O+H)^+^: 472.1151; found: 472.1135.

1-(4-chloro-3-(trifluoromethyl) phenyl)-3-(4-((2,3-dimethylphenyl) amino)-3,4-dihydroquinazolin-6-yl) urea (***7n***)

Yield: 81.6%; m.p. 203.3-204.6 ℃; compound purity: 98.270 %; aqueous solubility: soluble; IR (KBr) γ/cm^-1^: 3327, 3277, 3065, 1717, 1616, 1576, 1541, 1483, 1473, 1418, 1325, 1220, 1177, 1130, 829, 770, 662 cm^-1^; ^1^H NMR (400 MHz-DMSO-*d_6_*): δ 9.684 (s, 1H), 9.357 (s, 1H), 9.102 (s, 1H), 8.478-8.473 (d, *J* = 2.0 Hz, 1H), 8.299 (s, 1H), 8.214-8.209 (d, *J* = 2.0 Hz, 1H), 7.867-7.839 (dd, *J* = 2.0 Hz, 9.2 Hz, 1H), 7.736-7.627 (m, 3H), 7.126-7.101 (d, *J* = 10.0 Hz, 3H), 2.304 (s, 3H), 2.077-2.046 (d, *J* = 12.4 Hz, 3H); ^13^C NMR (100 MHz-DMSO-*d_6_*): δ 157.49, 152.19, 151.39, 144.51, 138.16, 136.34, 135.95, 135.72, 132.56, 130.88, 127.03, 126.54, 126.11, 125.80, 125.50, 125.30, 125.20, 124.33, 124.23, 123.07, 121.99, 121.38, 120.36, 117.64, 115.74, 115.68, 115.63, 114.05, 109.90, 18.99, 13.25. HRMS (ESI): m/z calculated for (C_24_H_19_ClF_3_N_5_O+H)^+^: 486.1308; found: 486.1292.

1-(4-fluorophenyl)-3-(4-(p-tolylamino)-3,4-dihydroquinazolin-6-yl) urea (***7o***)

Yield: 75.2%; m.p. 247.2-248.4 ℃; compound purity: 99.767 %; aqueous solubility: soluble; IR (KBr) γ/cm^-1^: 3290, 3254, 3065, 3032, 1647, 1624, 1558, 1541, 1508, 1420, 1356, 1211, 918, 833, 689 cm^-1^; ^1^H NMR (400 MHz-DMSO-*d_6_*): δ 9.684 (s, 1H), 8.938-8.934 (d, *J* = 1.6 Hz, 2H), 8.462-8.450 (t, *J* = 2.4 Hz, 2H), 7.904-7.877 (dd, *J* = 2.0Hz, 8.8 Hz, 1H), 7.749-7.682 (m, 3H), 7.545-7.510 (m, 2H),7.204-7.123 (m, 4H), 2.313 (s, 3H); ^13^C NMR (100 MHz-DMSO-*d_6_*): δ 162.89, 158.63, 157.30, 156.26, 152.83, 152.72, 145.77, 137.38, 136.81, 135.89, 135.87, 132.50, 128.77, 128.29, 126.35, 122.52, 120.09, 115.58, 115.40, 115.18, 110.47, 20.49; HRMS (ESI): m/z calculated for (C_22_H_18_FN_5_O+H)^+^: 388.1573; found:388.1581.

1-(4-((2,3-dimethylphenyl) amino)-3,4-dihydroquinazolin-6-yl)-3-(4-methoxy-phenyl) urea (***7p***)

Yield: 79.0%; m.p. 241.1-242.7 ℃; compound purity: 98.315 %; aqueous solubility: soluble; IR (KBr) γ/cm^-1^: 3346, 3281, 3184, 3130, 3063, 2951, 2831, 1680, 1639, 1622, 1602, 1570, 1556, 1512, 1462, 1431, 1375, 1360, 1325, 1302, 1231, 1038, 827, 768, 689 cm^-1^; ^1^H NMR (400 MHz-DMSO-*d_6_*):δ 9.614 (s, 1H), 8.828 (s, 1H), 8.680 (s, 1H), 8.396-8.391 (d, *J* = 2.0 Hz, 1H), 8.276 (s, 1H), 7.904-7.876 (dd, *J* = 2.0 Hz, 9.2 Hz, 1H), 7.712-7.690 (d, *J* = 8.8 Hz, 1H), 7.421-7.399 (d, *J* = 8.8Hz, 2H), 7.142-7.126 (d, *J* = 6.4 Hz, 3H), 6.902-6.879 (d, *J* = 9.2 Hz, 2H), 3.724 (s, 3H), 2.302 (s, 3H), 2.046 (s, 3H); ^13^C NMR (100 MHz-DMSO-*d_6_*): δ 158.53, 154.57, 153.12, 152.75, 145.47, 137.50, 137.43, 137.02, 133.65, 132.54, 128.18, 127.60, 126.06, 125.40, 125.36, 120.13, 115.15, 113.99, 110.05, 55.14, 20.13, 14.38; HRMS (ESI): m/z calculated for (C_24_H_23_N_5_O_2_+H)^+^: 414.1930; found: 414.1924.

1-(4-((4-bromophenyl) amino)-3,4-dihydroquinazolin-6-yl)-3-(4-methoxy-phenyl) urea (***7q***)

Yield: 87.6%; m.p. 267.1-268.2 ℃; compound purity: 97.615 %; aqueous solubility: soluble; IR (KBr) γ/cm^-1^: 3385, 3317, 2989, 2934, 2835, 1699, 1622, 1537, 1508, 1489, 1429, 1366, 1246, 1227, 1177, 825, 729, 683 cm^-1^; ^1^H NMR (400 MHz-DMSO-*d_6_*): δ 9.825 (s, 1H), 8.892 (s, 1H), 8.713(s, 1H), 8.519 (s, 1H), 8.475-8.470 (d, *J* = 2.0 Hz, 1H), 7.911-7.845 (m, 3H), 7.771-7.748 (d, *J* = 9.2 Hz, 1H), 7.574-7.552 (d, *J* = 8.8 Hz, 2H), 7.431-7.409 (d, *J* = 8.8 Hz, 2H), 6.910-6.888 (d, *J* = 8.8 Hz, 2H), 3.731 (s, 3H); ^13^C NMR (100 MHz-DMSO-*d_6_*): δ 156.98, 154.61, 152.75, 152.41, 145.71, 138.94, 137.86, 132.48, 131.12. 128.40, 126.45, 124.02, 120.17, 115.65, 114.95, 114.00, 109,86, 55.15; HRMS (ESI): m/z calculated for (C_22_H_18_BrN_5_O_2_+H)^+^: 464.0722; found: 464.0702.

1-(4-((4-fluorophenyl) amino)-3,4-dihydroquinazolin-6-yl)-3-(4-methoxy-phenyl) urea (***7r***)

Yield: 65.4%; m.p. 250.2-251.8 ℃; compound purity: 95.422 %; aqueous solubility: soluble; IR (KBr) γ/cm^-1^: 3298, 1651, 1628, 1614, 1578, 1553, 1528, 1508, 1425, 1387, 1360, 1300, 1248, 1171, 1032, 918, 829, 793, 768cm^-1^; ^1^H NMR (400 MHz-DMSO-*d_6_*): δ 9.813 (brs, 1H), 8.901 (s, 1H), 8.733 (s, 1H), 8.483 (s, 1H), 8.473-8.468 (d, *J* = 2.0 Hz, 1H), 7.912-7.40 (m, 4H), 7.440-7.418 (d, *J* = 8.8 Hz, 2H), 7.254-7.210 (t, *J* = 8.8 Hz, 2H), 6.916-6.893 (d, *J* = 9.2 Hz, 2H), 3.734 (s, 3H); ^13^C NMR (100 MHz-DMSO-*d_6_*): δ 159.54, 157.27, 157.15, 154.60, 152.76, 152.52, 145.43, 137.79, 135.67, 132.50, 128.20, 126.35, 124.47, 124.39, 120.17, 115.49, 115.04, 114.82, 113.99, 109.93, 55.13; HRMS (ESI): m/z calculated for (C_22_H_18_F_2_N_5_O_2_+H)^+^: 404.1522; found: 404.1511.

1-(4-methoxyphenyl)-3-(4-(p-tolylamino)-3,4-dihydroquinazolin-6-yl) urea (***7s***)

Yield: 65.8%; m.p. 259.5-260.1 ℃; compound purity: 98.141 %; aqueous solubility: soluble; IR (KBr) γ/cm^-1^: 3312, 3192, 3030, 2833, 1651, 1628, 1503, 1551, 1541, 1508, 1421, 1387, 1360, 1312, 1291, 1248, 1173,1030, 918, 827, 814, 791, 660 cm^-1^; ^1^H NMR (400 MHz-DMSO-*d_6_*): δ 9.684 (s, 1H), 8.856 (s, 1H), 8.717 (s, 1H), 8.455-8.435 (m, 2H), 7.903-7.876 (dd, *J* = 1.6 Hz, 9.2 Hz, 1H), 7.737-7.678 (m, 3H), 7.428-7.405 (d, *J* = 9.2 Hz, 2H), 7.202-7.181 (d, *J* = 8.4 Hz, 2H), 6.908-6.886 (d, *J* = 8.8 Hz, 2H), 3.728 (s, 3H), 2.312 (s, 3H); ^13^C NMR (100 MHz-DMSO-*d_6_*): δ157.28, 154.59, 152.77, 152.68, 145.53, 137.65, 136.81, 132.53, 128.78,128.20, 126.25, 122.51, 120.15, 115.59, 114.00, 110.13, 55.14, 20.51; HRMS (ESI): m/z calculated for (C_23_H_21_N_5_O_2_+H)^+^: 400.1773; found: 400.1775.

1-(4-chloro-3-(trifluoromethyl) phenyl)-3-(4-((2,3-dimethylphenyl) amino)-3,4-dihydroquinazolin-6-yl) urea (***7t***)

Yield: 75.2%; m.p. 241.9-242.5 ℃; compound purity: 98.558 %; aqueous solubility: soluble; IR (KBr) γ/cm^-1^: 3308, 3018, 2997, 2961, 2833, 1651, 1628, 1603, 1574, 1553, 1528, 1508, 1427, 1387, 1358, 1298, 1248, 1173, 1030, 918, 827, 793, 756, 671, 660 cm^-1^; ^1^H NMR (400 MHz-DMSO-*d_6_*): δ 9.635 (s, 1H), 8.822 (s, 1H), 8.695 (s, 1H), 8.414 (s, 2H), 7.888-7.861 (dd, *J* = 2.0 Hz, 8.8 Hz, 1H), 7.720-7.656 (m, 3H), 7.425-7.402 (d, *J* = 9.2 Hz, 2H), 6.976-6.954 (d, *J* = 8.8 Hz, 2H), 6.906-6.884 (d, *J* = 8.8 Hz, 2H), 3.774 (s, 3H), 3.727 (s, 3H); ^13^C NMR (100 MHz-DMSO-*d_6_*): δ 157.37, 155.66, 154.58, 152.82, 152.77, 145.55, 137,55, 132.53, 132.22, 128.23, 126.16, 124.36, 120.14, 115.47, 113.99, 113,56, 110.09, 55.18, 55.15; HRMS (ESI): m/z calculated for (C_23_H_21_N_5_O_3_+H)^+^: 416.1722; found: 416.1715.
